# Sustained and Efficient Ethanol Oxidation by Tethering Tunable N‐Donors Onto Integrated MXene/Pd‐Electrodes

**DOI:** 10.1002/advs.202513512

**Published:** 2025-10-13

**Authors:** Zhangxin Chen, Fan Jing, Alexandros Terzopoulos, Yanxian Jin, Kai Huang, Haichang Fu, Na Liu, Xianqiang Xiong, Binbin Yu, Dan Chen, Yang Xia, Dominic S. Wright

**Affiliations:** ^1^ Zhejiang Key Laboratory for Island Green Energy and New Materials Taizhou University Taizhou Zhejiang 318000 China; ^2^ Yusuf Hamied Department of Chemistry University of Cambridge Cambridge CB2 1EW UK; ^3^ School of Pharmaceutical and Chemical Engineering Taizhou University Taizhou Zhejiang 31800 China; ^4^ Taizhou Biomedical and Chemistry Industry Institute Taizhou Zhejiang 31800 China; ^5^ School of Chemistry and Molecular Engineering East China University of Science and Technology Shanghai 200237 China; ^6^ College of Materials Science and Engineering Zhejiang University of Technology Hangzhou 310014 China

**Keywords:** cycling stability, ethanol fuel cell, integrated electrode, MXene, Pd catalyst

## Abstract

The practical development of ethanol fuel cells as a future energy technology is currently set back by fundamental roadblocks (low cycling stability) and design shortcomings, e.g., poorly‐defined active sites and underexplored electrode integration strategies. Here, a rational design of 3D layered integrated electrodes is introduced for Pd‐catalysed ethanol oxidation, in which chemically grafted pyrrolic or picolinic amide linkers are employed to co‐anchor Pd nanoparticles on an MXene support via a “hand‐in‐hand” molecular coordination mode. This approach leads to good directional exposure of Pd (111) crystal surfaces through tailored ligand‐metal‐support interactions, creating well‐defined, more efficient Pd catalytic sites; thereby, the inherent limitations of the random distribution of the palladium surface sites can be overcome. The resulting system incorporating pyrrolic ligands exhibits excellent cycling stability (up to 10 000 cycles with 97.9% capacity retention), making it one of the most efficient ethanol oxidation catalysts reported. The robust Pd immobilisation after MXene functionalisation with the pyrrolic linkers allows achieving this performance with low Pd loadings, while calculations also indicate weaker adsorption of catalyst‐poisoning oxidation intermediates. This new, modular approach provides a pathway to more efficient electrocatalytic systems for real‐world applications in ethanol fuel cells.

## Graphical abstract

Three novel 3D layered integrated electrodes for Pd‐catalysed ethanol oxidation are developed; their innovative design features N‐heterocyclic amide linkers grafted on an MXene, co‐anchoring Pd nanoparticles via “hand‐in‐hand” coordination. The resulting well‐defined Pd (111) catalytic sites help achieve extremely stable long‐term cycling with sustained efficiency.

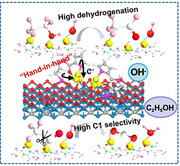

## 1 Introduction

Amidst the continuously increasing global demand for alternative energy technologies, ethanol fuel cells stand out as a particularly promising and greener high‐efficiency option; their possible applications include portable electronics, vehicles, and stationary power generation.^[^ ^1^, ^2^ ^]^ Despite their potential, ethanol fuel cells face several challenges, such as issues of durability and integration. Two major problems related to the chemical properties of the fuel cell materials are: a) the electrocatalytic activity of metal catalysts, most commonly the noble metals platinum (Pt) and palladium (Pd), which are prone to deactivation due to poisoning; and b) the electrode cycling stability, which can be hindered similarly by the formation of by‐products and catalyst deactivation.^[^ ^3^, ^4^ ^]^

One common way to improve the activity of ethanol or methanol oxidation Pt or Pd‐based electrocatalysts is the modulation of the metal's electronic structure; this can be achieved through introducing strong metal‒support interactions by doping heteroatoms into the catalyst support in integrated electrodes.^[^ ^5^, ^6^, ^7^ ^]^ Such modification of the local coordination environment around the catalytically active metal can have an immense influence on prolonging ethanol fuel cell lifetimes and performance.^[^ ^8^ ^]^ Various studies on Pd‐loaded systems have examined the introduction of nitrogen‐containing dopants for these purposes, owing to the strength of Pd···N interactions and their effect on the density of states of Pd systems.^[^ ^9^, ^10^ ^]^ Nonetheless, multiple types of N‐doped sites usually co‐exist embedded on the support, fulfilling different functions; as an illustration, in N‐doped graphene‐based supports, graphitic N sites enhance the graphene's electrical conductivity, whereas pyridinic (terminal) N sites promote the attachment and dispersion of the metallic nanoparticles on the support.^[^ ^7^ ^]^

In parallel to advances in doping, an emerging development within electrocatalysis has been the selection of MXenes as noble metal catalyst supports.^[^ ^8^, ^9^, ^10^ ^]^ MXenes are 2D inorganic materials consisting of ultrathin layers of transition metal carbides or nitrides, possessing excellent electrical conductivity and electrochemical activity, tuneable properties and abundant surface functionalities that aid the immobilisation of metallic nanoparticles.^[^ ^11^, ^12^ ^]^ The earliest MXene to be utilised as an electrocatalytic support was Ti_3_C_2_T*_x_* (where T represents the randomly distributed terminal groups, viz. ─OH, ─O^−^, ─F),^[^ ^13^ ^]^ and it continues to dominate the latest research in the field due to its facile synthesis and high Pd/Pt‐anchoring ability.^[^ ^14^, ^15^ ^]^ A large number of these investigations have focused specifically on the application of Ti_3_C_2_T*_x_*‐supported Pd nanoparticles (Pd^0^ NPs) in direct methanol or ethanol fuel cells.^[^ ^17^, ^18^ ^]^ In our previous work, we wanted to probe whether the above‐mentioned benefits of heteroatom doping could be applied to these increasingly popular MXene supports, and hence prepared a series of N‐doped and B/N co‐doped Ti_3_C_2_T*_x_*‐supported Pd catalysts; the resulting systems indeed showed noticeably higher activity for the electrocatalytic oxidation of ethanol over commercial Pd/C.^[^ ^8^, ^9^ ^]^

**Figure 1:**
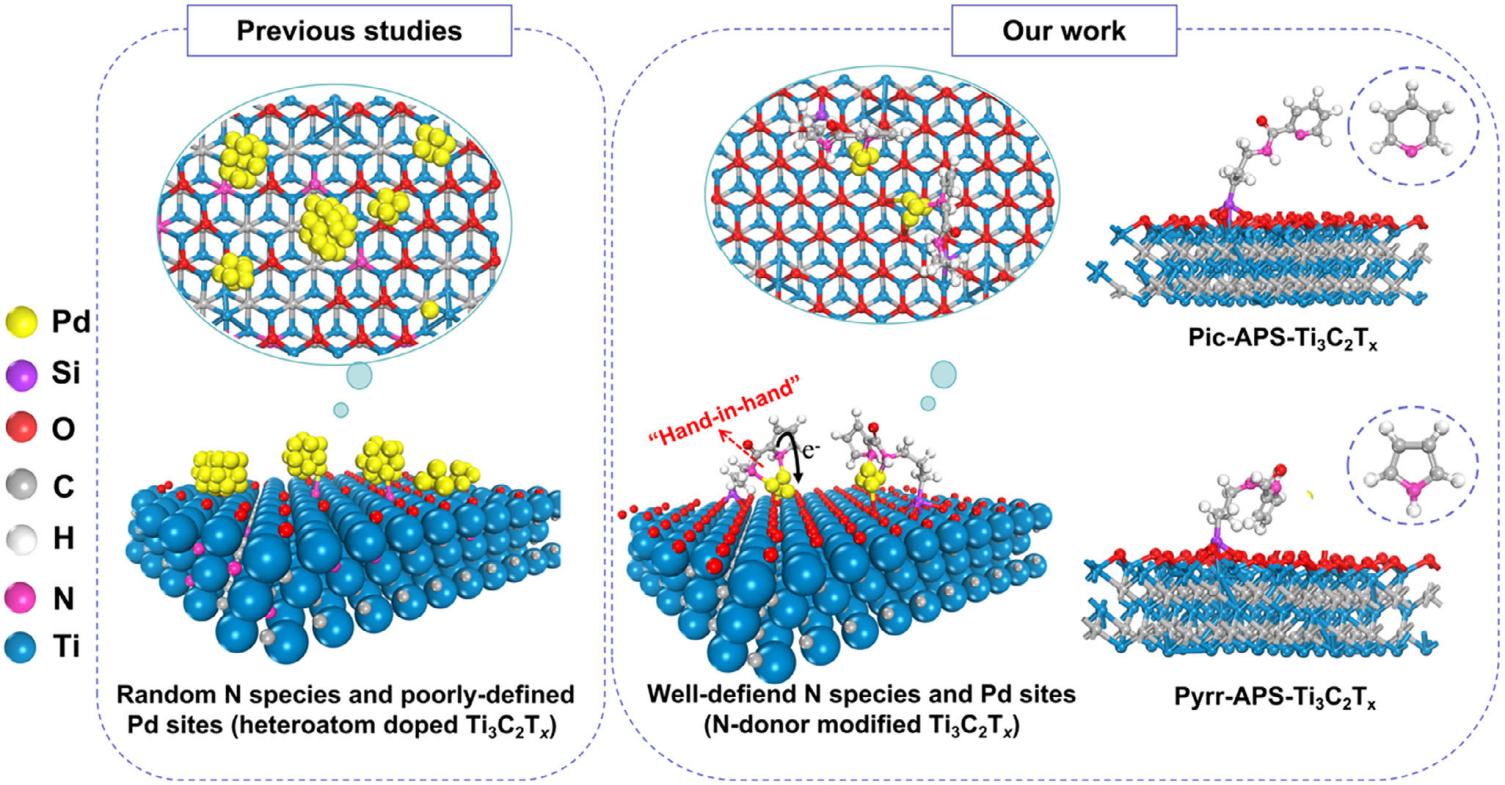
Comparison of previously reported doped Ti_3_C_2_T*_x_*‐supported systems for Pd‐based fuel cell electrocatalysts (left) with the novel N‐donor‐functionalised Ti_3_C_2_T*_x_* catalysts prepared in this study (right), including a schematic representation of the pyridine‐ and pyrrole‐based catalytic supports.

In this study, we report a novel strategy for the design of an MXene‐based fuel cell electrocatalyst support, wherein the surface of the Ti_3_C_2_T*_x_* nanosheet has been modified by a two‐step chemical functionalisation with discrete N‐donor ligands instead of uncontrolled single‐heteroatom doping (left part of **Figure** **1**). The hydroxyl surface terminations (─T = ─OH) of Ti_3_C_2_T*_x_* are in equilibrium with their deprotonated form (─T = ─O^−^), making the surface negatively charged over a wide pH range (*≈*4–12)^[^ ^19^ ^]^ and thereby facilitating surface functionalisation via etherification reactions.^[^ ^20^, ^21^ ^]^ Such modifications have been demonstrated previously by condensation of the surface hydroxy‐/oxido‐terminations with trialkoxysilane coupling reagents, RSi(OR)_3_, where ‒R is an aliphatic hydrophobic (long‐chain alkane) or hydrophilic (H_2_N‐containing) side group.^[^ ^23^, ^24^ ^]^ Nonetheless, in the prior literature, these silane‐modified MXenes have been used either on their own or as nanocomposites in combination with polymeric layers exclusively as high‐performance membranes or drug/solvent carrier systems.^[^ ^22^, ^23^, ^24^ ^]^ To the best of our knowledge, this work presents the first example of both: (*i*) a two‐step small‐molecule functionalisation routine of the MXene, i.e., the chemical post‐modification of the side‐group of the surface‐grafted silane to afford a well‐defined ligand site; and (*ii*) the application of a surface‐functionalised (instead of doped) MXene for electrocatalysis.

The conceptual benefits of this strategy are twofold. The first advantage is that substituting single‐heteroatom dopants for discrete N‐donor ligands on the surface of the electrocatalytic support can help clarify the previously mentioned incertitude on the nature of the active sites interacting with the Pd catalysts. By introducing two small nitrogen‐containing molecules containing either a pyridine or pyrrole ring (right part in Figure 1) with predictable electronic push‐pull properties of the N‐donating moiety, we have developed a model system employing heterocyclic amidic linkers to co‐anchor palladium nanoparticles on the surface or in the interlayer spaces of the MXene support via “hand‐in‐hand” molecular coordination (i.e., within the specific‐size cavity formed by the donor moieties of the tethered ligand and MXene surface sites). This design principle has been used to explore the relationship between surface‐active nitrogen species and the electrocatalytic properties of the palladium catalyst—the Pd nanoparticles are influenced by both surface (support‐embedded) oxygen and the tethered N‐donors, resulting in more pronounced electron transfer from the functional organic layer toward the anchored Pd species.

The second benefit of the MXene surface functionalisation is the prevention of some of the issues associated with the structural layout of the electrode. Electrodes prepared in the traditional manner (involving glassy carbon or carbon cloth substrates) are plagued by poor adhesion between the substrate material and the active metal catalyst, resulting in dissociation of some active material during processing and a subsequent reduction in catalyst lifetime. The use of an efficient immobilisation matrix, such as a Ti_3_C_2_T_x_ sheet, enhanced with the ligating N‐donors, circumvents this problem. Concurrently, grafting organic spacers on the surface can help alleviate the aggregation of the 2D nanosheets, which is known to lower electrocatalytic performance.^[^ ^21^, ^24^ ^]^ We have taken advantage of these structural features of the functionalised MXene in our electrode design, which was based on the self‐assembly of the 2D material into a 3D network, aiming to increase the electrochemically active area and accelerate reaction kinetics.^[^ ^25^ ^]^ The resulting electrodes with the two novel catalysts (either with pyridine‐ or pyrrole‐based donors, see Figure 1 for labelling convention) show superior cycling stability compared to Pd/C systems, with the 3D pyrrole‐based catalyst exhibiting the highest retention (97.9%) after 10000 cycles. Investigations using in situ Raman spectroscopy, differential charge density, adsorption energy and theoretical density of states (DOS) calculations of the catalyst and its integrated electrode are used to explore the effects of the electron interactions between Pd and the modified Ti_3_C_2_ support, providing insights into the impact of electron‐donating abilities on electrocatalytic properties during the ethanol oxidation reaction (EOR).

## 2 Results and Discussion

### 2.1 Design of the Functionalised MXene Catalyst

**Scheme 1:**
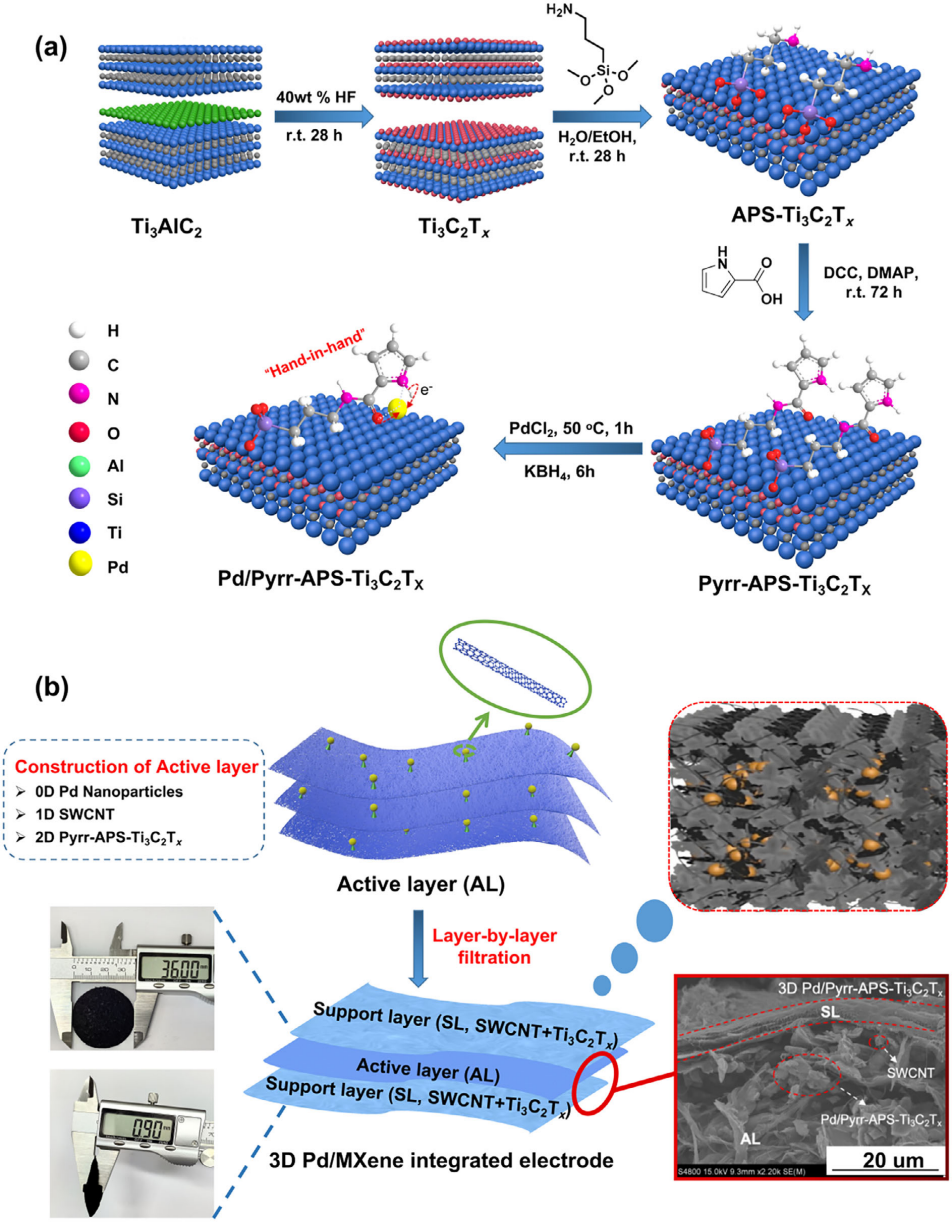
a) Synthetic route for the preparation of the pyrrole‐ring containing catalyst by reacting the Ti_3_C_2_T*_x_* nanosheet with APTMS to afford the aminopropylsiloxy‐functionalised MXene (“APS‐Ti_3_C_2_T*_x_*”) followed by esterification with 2‐pyrrolecarboxylic acid, producing the (1H‐pyrrole‐2‐carboxamido)propyl‐siloxy‐functionalised MXene (“Pyrr‐APS‐Ti_3_C_2_T*_x_*”), which is then loaded with Pd. An analogous route with picolinic acid produces the (picolinamido)propylsiloxy‐functionalised MXene (“Pic‐APS‐Ti_3_C_2_T*_x_*”) shown on the bottom left of Figure 1. b) Schematic depiction of the assembly of the 3D Pd/Pyrr‐APS‐Ti_3_C_2_T_x_ integrated electrodes. This assembly can be used for any catalyst material, such as 3D Pd/Pic‐APS‐Ti_3_C_2_T_x,_ 3D Pd/C, etc. (crimson insert: SEM image of the layered structure).

The novel MXene catalyst supports were obtained as powders starting from Ti_3_C_2_T*_x_* prepared by the standard method (see Experimental Section), which was then functionalised by condensation with (3‐aminopropyl)trimethoxysilane (APTMS), followed by Steglich esterification to introduce the pyridine‐ (Pic‐APS‐Ti_3_C_2_T*_x_*) and pyrrole‐containing (Pyrr‐APS‐Ti_3_C_2_T*_x_*) amidic moieties on the surface of the MXene (**Scheme** **1**a). The catalytically active Pd^0^ NPs were formed in situ from the reduction of a Pd(II) salt using KBH_4_; the resulting Pd‐loaded MXene was either used as a powder catalyst (as obtained) or assembled into a 3D integrated electrode in a layer‐by‐layer fashion (Scheme 1a,b). The layered integrated electrodes were constructed with a top and bottom support layer (SL) and an intermediate active layer (AL) containing the functionalised MXene, as shown in Scheme 1b. The SLs were chosen to have porous structures in order to facilitate transport of electrolyte ions and promote interfacial reactions in contact with the AL (Figure S1, Supporting Information). In total, four 3D Pd/functionalised‐Ti_3_C_2_T*_x_* integrated electrodes and four powder electrodes were prepared. Their Pd loadings, as determined by ICP spectrometry, are given in Table S1 (Supporting Information).

### 2.2 Characterisation of the Materials

**Figure 2:**
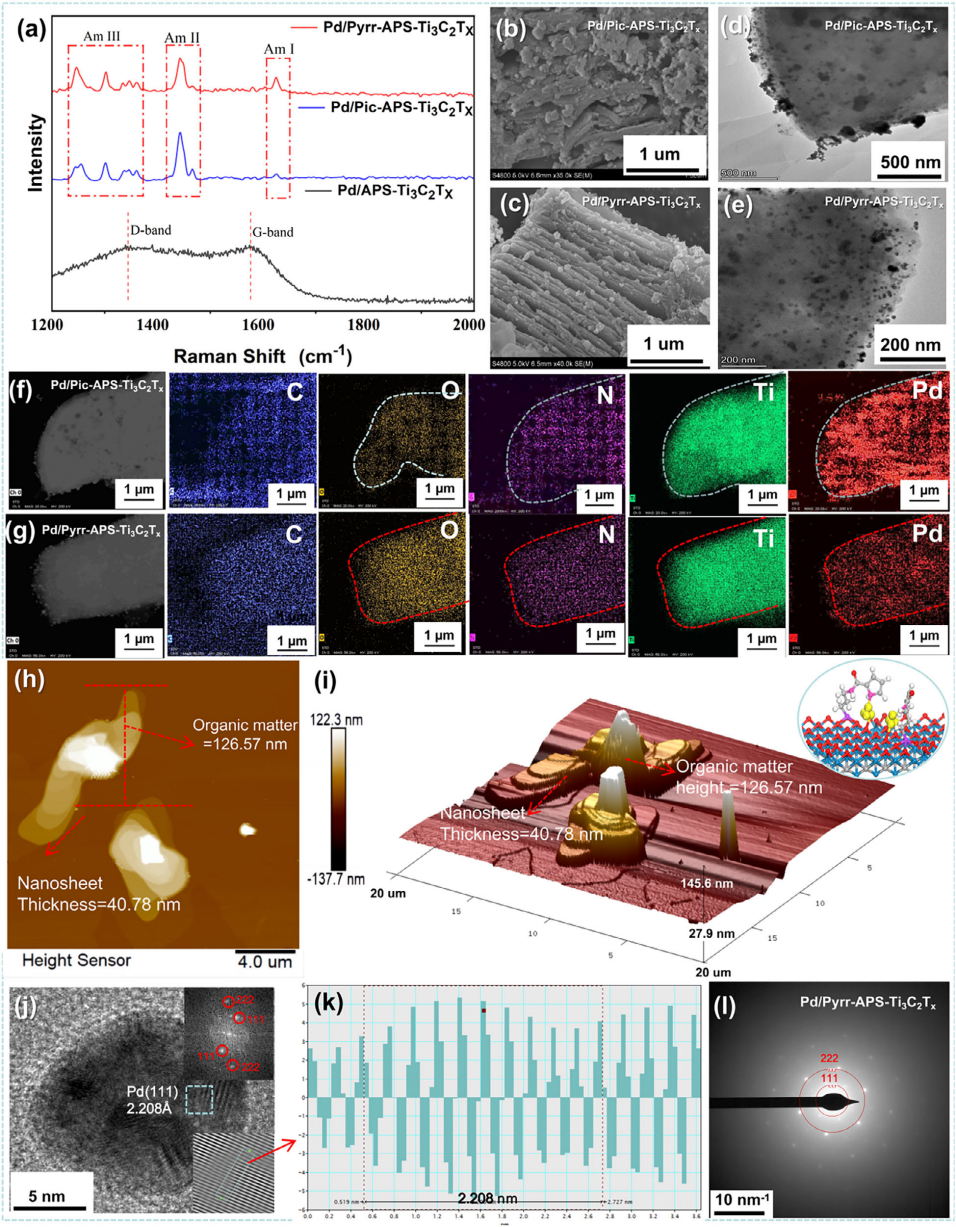
Comparison of: a) Raman spectra of the three Pd‐loaded functionalised MXenes; b,c) SEM micrographs; d,e) TEM micrographs of Pd/Pic‐APS‐Ti_3_C_2_T*_x_* and Pd/Pyrr‐APS‐Ti_3_C_2_T*_x_*; f,g) TEM elemental mapping (for O, C, Ti, Si, N and Pd) of Pd/Pic‐APS‐Ti_3_C_2_T*_x_* and Pd/Pyrr‐APS‐Ti_3_C_2_T*_x_*; h) 2D topographic AFM mapping; i) 3D rendering of the AFM mapping; j) High resolution TEM (HR‐TEM) image—insert: fast Fourier transform (FFT) pattern of the HR‐TEM image; k) Pd (111) crystal size measurement from the HR‐TEM image; l) SAED patterns of Pd/Pyrr‐APS‐Ti_3_C_2_T*_x_*.

The morphologies and microstructures of the Pd/functionalised‐Ti_3_C_2_T*_x_* powder composites and their integrated electrodes, as well as the local coordination environments for Pd atoms therein, were investigated by Raman spectroscopy, scanning electron microscopy (SEM), atomic force microscopy (AFM) and transmission electron microscopy (TEM) coupled with selected area diffraction (SAED) measurements (**Figure** **2**).

Starting with the Raman measurements, the strength of the interaction between the Pd nanoparticles and N‐donating linkers in Pic‐APS‐Ti_3_C_2_T*_x_* and Pyrr‐APS‐Ti_3_C_2_T*_x_* becomes evident from the Raman spectra of the Pd‐doped functionalised substrates (Figure 2a). Pd/APS‐Ti_3_C_2_T*_x_* (i.e., the aminosilane‐treated substrate with no amide linker) exhibited only the multiple broad peaks of Ti_3_C_2_T*_x_* between 1200 and 1700 cm^−1^, which can be ascribed tentatively to the G‐band and D‐band of amorphous carbon formed by the partial lattice damage of MXene*_._* In contrast, the spectra of Pd/Pic‐APS‐Ti_3_C_2_T*_x_* and Pd/Pyrr‐APS‐Ti_3_C_2_T*_x_* showed the typical peaks expected of the amide group and the heterocyclic rings (pyridine and pyrrole, respectively). The intensity of the peak belonging to the amide C═O stretch located at 1660 cm^−1^ (Am I in Figure 2a) was much stronger for Pd/Pyrr‐APS‐Ti_3_C_2_T*_x_* than for Pd/Pic‐APS‐Ti_3_C_2_T*_x_*, indicating that in the former case the amide carbonyl is involved in Pd coordination. Disparately, among the multiple peaks in the 1250‒1360 cm^−1^ region (attributed to the breathing vibrations of the pyrrole or pyridine rings; Am III in Figure 2a), there is a pronounced split peak at 1250 cm^−1^ in Pd/Pic‐APS‐Ti_3_C_2_T*_x_* which is probably indicative of Pd coordination to the pyridine N. These results indicate that for the pyrrole‐based ligand, coordination happens through both the pyrrolic nitrogen and the amidic oxygen, which helps co‐anchor palladium in a “hand‐in‐hand” mode (between the effectively bidentate linker and the oxygen donor‐rich MXene surface) in Pd/Pyrr‐APS‐Ti_3_C_2_T*_x_*, whereas for the picolinamide ligand, only the pyridinic nitrogen interacts strongly (other than the MXene support itself) with the palladium in Pd/Pic‐APS‐Ti_3_C_2_T*_x_*.

Regarding the morphology of the materials as seen with SEM, the starting MXene (unfunctionalised Ti_3_C_2_T*_x_*) exhibited the typical exfoliated morphology after HF etching (Figure S2a, Supporting Information). Among the three Pd‐loaded functionalised MXenes, the one with pyrrole linkers (Pd/Pyrr‐APS‐Ti_3_C_2_T*_x_*) exhibited uniform grafting of the Pd nanoparticles on its surface, whereas Pd/Pic‐APS‐Ti_3_C_2_T*_x_* and the unesterified Pd/APS‐Ti_3_C_2_T*_x_* had a much more irregular morphology (Figure 2b,c; Figure S2b, Supporting Information). More detailed investigation with TEM showed that Pd/Pyrr‐APS‐Ti_3_C_2_T*_x_* had the highest and most uniform distribution of Pd particles loaded on Ti_3_C_2_T*_x_* (Figure 2e), contrasting with the low distribution of Pd particles on the MXene support in Pd/APS‐Ti_3_C_2_T*_x_* (Figure S3, Supporting Information) and high aggregation of palladium on the MXene edge in Pd/Pic‐APS‐Ti_3_C_2_T*_x_* (Figure 2d; Figure S4, Supporting Information). TEM mapping in the Pd/Pyrr‐APS‐Ti_3_C_2_T*_x_* catalyst showed clearly that the distribution of Pd nanoparticles is more consistent with the distribution of nitrogen and oxygen (Figure 2g) compared with the case in Pd/Pic‐APS‐Ti_3_C_2_T*_x_* (Figure 2f). This concurs with the Raman spectra to support that the Pd nanoparticles are indeed tethered by both the pyrrolic‐APTMS linkers and the surface oxygen on the surface or within the interlayer space of Ti_3_C_2_T*_x_* (Figure S4, Supporting Information). An AFM measurement of the Pd/Pyrr‐APS‐Ti_3_C_2_T*_x_* system revealed that it contained a Ti_3_C_2_ nanosheet with a thickness of 40.78 nm and an organic layer (linked to Pd nanoparticles) with a total thickness of 126.57 nm. Notably, the Ti_3_C_2_T*_x_* support in reality exhibits a multilayer structure, wherein Pd nanoparticles are influenced simultaneously by support‐embedded oxygen atoms and grafted nitrogen donors on the surface or in the interlayer spaces of Ti_3_C_2_T*_x_*. (Figure 2h,i). Selected area diffraction (SAED) experiments revealed an unclear and disordered polycrystalline structure for both Pd/APS‐Ti_3_C_2_T*_x_* and Pd/Pic‐APS‐Ti_3_C_2_T*_x_*, illustrating the randomly exposed Pd crystalline surfaces in these two catalysts (Figure S5, Supporting Information). In contrast, Pd/Pyrr‐APS‐Ti_3_C_2_T*_x_* displayed a distinct crystalline Pd (111) and Pd (222) structure according to HRTEM and SAED (Figure 2j–l). The presence of the Pd (222) surface reflects high ordering and small particle size in this system, hinting at the presence of highly active electrocatalytic sites. This difference can be attributed to the specific coordination mode of the pyrrole amide ligand (involving both the pyrrolic N and amidic O, vide supra), allowing directional exposure of the (111) lattice plane.

Powder X‐ray diffraction (PXRD) analyses were performed for all three Pd‐loaded functionalised MXenes and are shown in Figure S6 (Supporting Information). Both Pd/Pic‐APS‐Ti_3_C_2_T*_x_* and Pd/APS‐Ti_3_C_2_T*_x_* exhibited weak peaks at 39.1°, 45.2°, and 68.7° corresponding to the (111), (200), and (220) characteristic lattice planes of Pd metal, respectively. Surprisingly, apart from these typical peaks for Pd lattices, Pd/Pyrr‐APS‐Ti_3_C_2_T*_x_* showed the sharp peak of (111) lattice at 39.6° and a new peak at 86.4° assigned to the (222) lattice (of face‐centred cubic Pd metal as per PDF card № 46‐1043)^10^. These results are consistent with the SAED patterns, indicating the existence of well‐exposed, highly‐ordered (111) Pd lattice planes in Pd/Pyrr‐APS‐Ti_3_C_2_T*_x_*.

**Figure 3:**
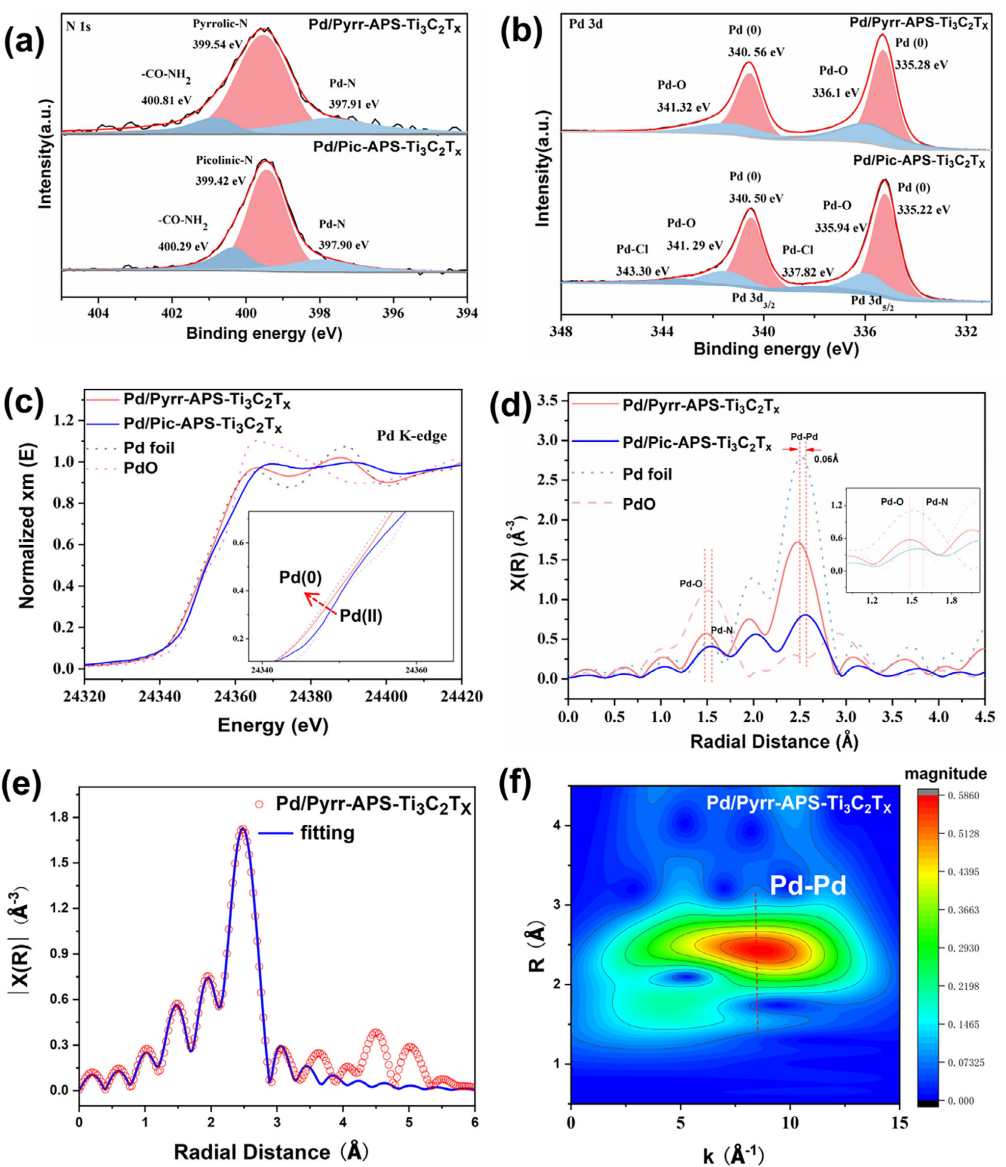
XPS elemental fitting of: a) N 1s and b) Pd 3d for Pd/Pyrr‐APS‐Ti_3_C_2_T*_x_* (above) and Pd/Pic‐APS‐Ti_3_C_2_T*_x_* (below); c) Comparison of the Pd K‐edge XANES spectra; d) Comparison of Pd K‐edge EXAFS, shown in *k* ^2^ weighted R‐space; e) EXAFS *k* space fitting curve and f) wavelet transform for the EXAFS of Pd/Pyrr‐APS‐Ti_3_C_2_T*_x_*.

X‐ray photoelectron spectroscopy (XPS) was employed to study the affinities between pyrrole‐ and pyridine‐modified Ti_3_C_2_ matrices and Pd nanoparticles. In the survey spectrum, the Ti 2p peak can be fitted well to only one Ti species (i.e., having all Ti environments participating in C─Ti─O bonds) located at 464 eV (Ti 2p_1/2_) and 458.5 eV (Ti 2p_3/2_) in all of the catalysts prepared (Figure S7, Supporting Information, left). They also all possessed two similar characteristic Si 2p fitting peaks, which are located at ≈102 eV and 101 eV (Figure S7, Supporting Information, right) and correspond to Si─OCH_3_ and Si─CH_2_ bonds, respectively—the former indicating incomplete condensation of APTMS on the base Ti_3_C_2_T*_x_* layer. Most interestingly, three types of 1s N species were distinguished in Pd/Pyrr‐APS‐Ti_3_C_2_T*_x_*: CO‐NH (amidic N: N1), pyrrolic N (N2), and Pd···N (N3). For the pyridine‐containing catalyst, Pd/Pic‐APS‐Ti_3_C_2_T*_x_*, the 1s N peak was fitted analogously to CO‐NH (N1), pyridinic N (N2), and Pd···N (N3) (**Figure** **3**a). Pd‐Pyrr‐APS‐Ti_3_C_2_T_x_ exhibits a higher binding energy of the amide unit (400.81 eV) compared to that (400.29 eV) in Pd‐Pic‐APS‐Ti_3_C_2_T_x_, which can be attributed to the more pronounced electron transfer from the amide and pyrrole linker toward the anchored Pd species. This increased electron density on Pd subsequently induces a stronger back‐donation effect to the CO─NH_2_ group, which stabilises its core energy levels and thus leads to a higher binding energy for the N 1s electron in the CO─NH_2_ moiety. These results strongly support the findings from Raman spectroscopy, indicating the effective anchoring of the Pd nanoparticles with the nitrogen‐donor site (N3) in Pd/Pyrr‐APS‐Ti_3_C_2_T*_x_* and Pd/Pic‐APS‐Ti_3_C_2_T*_x_*. The Pd 3d spectra of the three catalysts consisted of a high‐energy band (Pd 3d_3/2_) and a low‐energy band (Pd 3d_5/2_), located at *ca* 335.3 and 336.1 eV, corresponding to Pd^0^ and Pd─O, respectively. Pd─O peaks appeared in all three catalyst materials, suggesting that Pd nanoparticles form bonds with the oxygen atoms on the surface or in the interlayer spaces of Ti_3_C_2_T*_x_*.^[^ ^28^ ^]^ Notably, a pair of new peaks characteristic of a Pd─Cl bond located at 342/338 eV was also present in both Pd/Pic‐APS‐Ti_3_C_2_T*_x_* and Pd/APS‐Ti_3_C_2_T*_x_* but absent in Pd/Pyrr‐APS‐Ti_3_C_2_T*_x_* (Figure 3b; Figure S8, Supporting Information). This observation can be attributed to incomplete reduction of the PdCl_2_ precursor during the reductive deposition step (last reaction in Scheme 1a), thereby retaining some Pd─Cl bonds. In addition, the characteristic peaks for the Pd─N bond could not be clearly observed in the Pd 3d region of all three catalysts, which may be due to the weakness of these signals compared with other Pd peaks. The whole XPS spectrum and also the corresponding elemental compositions of the three catalysts are shown in Figure S9 and Table S2 (Supporting Information). The Pd content in Pd/Pyrr‐APS‐Ti_3_C_2_T*_x_* (3.5 wt%) was much lower than in Pd/Pic‐APS‐Ti_3_C_2_T*_x_* (10.5 wt%) and Pd/APS‐Ti_3_C_2_T*_x_* (7.6 wt%); nonetheless, the much better electrocatalytic performance (vide infra) found for Pd/Pyrr‐APS‐Ti_3_C_2_T*_x_* may be due to a combination of the strong electronic interaction and its better‐ordered morphology in comparison to the other functionalised MXene catalysts.

To explore further the Pd···N interactions at the atomic level, X‐ray absorption near‐edge structure (XANES) and extended X‐ray absorption fine structure (EXAFS) studies were carried out. The Pd K‐edge in the XANES spectrum of Pd/Pic‐APS‐Ti_3_C_2_T*_x_* was close to that of PdO, while the energy position for Pd/Pyrr‐APS‐Ti_3_C_2_T*_x_* was close to that of Pd foil (Figure 3c).^[^ ^27^ ^]^ As can also be seen from the EXAFS of these catalysts (Figure 3d), a typical Pd‒Pd bond distance (2.58 Å) similar to that in Pd foil was observed in Pd/Pyrr‐APS‐Ti_3_C_2_T*_x_*, whereas Pd/Pic‐APS‐Ti_3_C_2_T*_x_* has a Pd─Pd radial distance which was 0.06 Å greater.^[^ ^30^ ^]^ These results demonstrate the dominant Pd(0) metallic state in the interaction with the pyrrole‐functionalised support. In addition, Pd/Pyrr‐APS‐Ti_3_C_2_T*_x_* showed a main strong peak at ≈1.48 Å, assigned tentatively to a Pd─O═C bond (differing slightly from the ≈1.50 Å Pd─O bond in a standard PdO sample) and a weak shoulder corresponding to Pd─N at ≈1.6 Å, while Pd/Pic‐APS‐Ti_3_C_2_T*_x_* exhibited a weak Pd‒N peak at ≈1.6 Å. The coordination environments of the Pd and N species were examined more closely by conducting quantitative EXAFS curve fitting, which revealed detailed information about back‐scattering paths and the extent of metal coordination (Figure 3e; Figures S10, S11, Table S3, Supporting Information). Notably, the coordination radial distances of the Pd─N and Pd─O bonds were very similar, leading to serious peak overlap that renders them difficult to distinguish. For Pd/Pic‐APS‐Ti_3_C_2_T*_x_*, the fitted curve can be divided into the three sample‐averaged coordination environments containing Pd‒Pd bonds (≈2.74 Å), Pd─O/N bonds (≈2.00 Å) and Pd─Cl bonds (≈2.25 Å). Per *contra*, Pd─Cl bonds could not be observed in Pd/Pyrr‐APS‐Ti_3_C_2_T*_x_*, which is in good agreement with the XPS results (Figure 3b). Additionally, the average coordination numbers of the Pd─Pd (≈7.0) and Pd─O/N (≈1.6) in Pd/Pyrr‐APS‐Ti_3_C_2_T*_x_* were much higher than those in Pd/Pic‐APS‐Ti_3_C_2_T*_x_* (Pd‒Pd: ≈2.5; Pd─O/N: ≈0.2), suggesting a stronger electronic interaction (presumably aided by the more uniform nanoparticle distribution) in the pyrrole‐based catalyst. Furthermore, the wavelet‐transformed EXAFS of Pd/Pyrr‐APS‐Ti_3_C_2_T*_x_* exhibited the main intensity maximum at ≈8.5 Å^−1^, which was attributed to Pd─Pd scattering only, while for Pd/Pic‐APS‐Ti_3_C_2_T*_x_* two signals were present at ≈8.5 Å^−1^ (major) and 5.0 Å^−1^ (rather minor intensity), corresponding to Pd─Pd and weak Pd─Cl scattering, respectively (Figure 3f; Figure S11, Supporting Information). This confirms that complete reduction and stabilisation of Pd(0) were only effected with the pyrrolic ligand, as also seen in the aforementioned EXAFS and XPS data (Figure 3b; Table S3, Supporting Information). In conclusion, it appears that the electronics of the Pd···support interactions in Pd/Pyrr‐APS‐Ti_3_C_2_T*_x_* are particularly favourable and are therefore likely to enhance the electrocatalytic properties of that catalyst for EOR, as is demonstrated in the subsequent section.

### 2.3 Electrocatalytic Performance Evaluation

**Figure 4:**
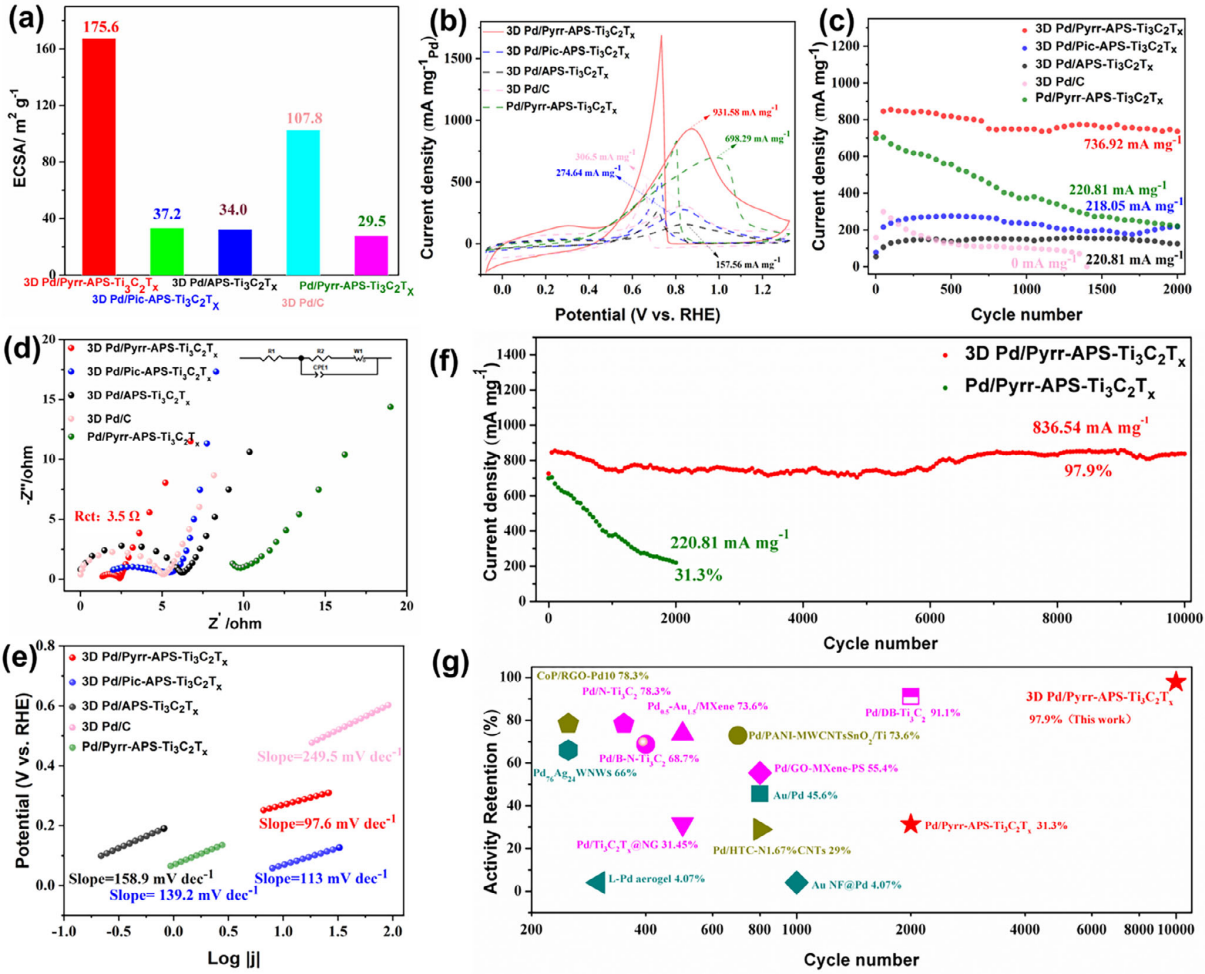
a) Comparison of the specific ECSA values for the four 3D integrated electrodes and powder 3D Pd/Pyrr‐APS‐Ti_3_C_2_T*_x_*; b) CV curves for these systems in 1 m CH_3_CH_2_OH/KOH (scan rate: 50 mV s^−1^); c) Current densities for these systems in 1 m CH_3_CH_2_OH/KOH over 2000 cycles; d) EIS *Z*′/‐*Z*″ plot for these systems; e) Tafel slopes for these systems; f) Long‐term durability over 10 000 cycles for 3D Pd/Pyrr‐APS‐Ti_3_C_2_T*_x_* and 2000 cycles for Pd/Pic‐APS‐Ti_3_C_2_T*_x_*; g) Scatterplot comparing electroactivity retention versus cycling of different catalysts in previous articles (colour‐coded by support type) with the herein‐reported Pd/Pyrr‐APS‐Ti_3_C_2_T*_x_*.^[^ ^29^, ^30^, ^31^, ^32^, ^33^, ^34^, ^35^, ^36^, ^37^, ^38^ ^]^

The four 3D catalyst integrated electrodes (3D Pd/Pyrr‐APS‐Ti_3_C_2_T*_x_*, 3D Pd/Pic‐APS‐Ti_3_C_2_T*_x_*, 3D Pd/APS‐Ti_3_C_2_T*_x_* and 3D Pd/C) were assembled with layer‐by‐layer filtration and then used as the working electrode in a three‐electrode cell (where Pt wire was used as the counter electrode and a Hg/HgO electrode as the reference electrode in a 1 m KOH solution at a scan rate of 50 mV s^−1^). The *E*_RHE_ can be obtained by the equation: *E*_RHE_ = *E*_test_ + *E*°_Hg/HgO_ + 0.059×pH. Between these four powder catalyst electrodes, 3D Pd/Pyrr‐APS‐Ti_3_C_2_T*_x_* showed the highest current density for the reduction peaks (≈0.7 V vs RHE), exhibiting an excellent reduction peak area of Pd^II^ oxide (PdO) in alkaline media. On the basis of this reduction peak area, the electrochemically active surface area (ECSA) can be calculated according to the equation *ECSA* = *Q*/(*m*_Pd_ × 0.405), where *m*_Pd_ is the Pd loading on the electrode (in mg) that is measured by ICP, *Q* is the Coulombic charge (in mC) calculated by integrating the reduction peak area of PdO, and 0.405 represents the charge required for the reduction of the PdO monolayer (in mC cm^−2^). Performing this calculation, the ECSAs of the four powder catalyst electrodes (Pd/Pyrr‐APS‐Ti_3_C_2_T*_x_*, Pd/Pic‐APS‐Ti_3_C_2_T*_x_*, Pd/APS‐Ti_3_C_2_T*_x_* and Pd/C) were measured to be 29.5, 5.6, 1.2, and 26.2 m^2^ g^−1^, respectively (Figure S12 and Table S4, Supporting Information). For the four corresponding 3D integrated electrodes, the redox peaks of absorption and desorption of hydrogen (0.3/0.1 V vs RHE) were much more striking than those of the powder catalyst electrodes, resulting in appreciably higher ECSAs (Figure S13, Supporting Information). The ECSAs calculated for the corresponding 3D integrated electrodes were 175.6, 37.2, 34.0, and 107.8 m^2^ g^−1^ (**Figure** **4**a; Table S4, Supporting Information), indicating more active Pd sites and being consistent with the SEM results (Figure S1, Supporting Information).

The electrocatalytic properties of the catalysts were tested under simulated alkaline conditions in the presence of ethanol to measure the oxidation peak located at ≈0.95 V corresponding to the continuous dehydrogenation of ethanol and formation of absorbed intermediates (*e.g*., CO_(ads)_ or CH_3_CO_(ads)_). The mass activity of powder Pd/Pyrr‐APS‐Ti_3_C_2_T*_x_* (698.29 mA mg^−1^) was considerably higher than that of powder Pd/Pic‐APS‐Ti_3_C_2_T*_x_* (102.1 mA mg^−1^) and Pd/C (185.38 mA mg^−1^) in terms of the maximum ethanol oxidative current densities (Figure S14, Supporting Information). Following a similar trend, 3D Pd/Pyrr‐APS‐Ti_3_C_2_T*_x_* revealed the highest mass activity compared to all of the powder and 3D integrated electrodes, again hinting at the strength of the Pd···support interaction (Figure 4b), as well as highlighting the benefits of the unique 3D porous structure of the integrated electrodes (Figure S1, Supporting Information). The exact same phenomenon was observed when we prepared Pt‐loaded analogues of the pyrrole‐functionalised catalyst (both as powder Pt/Pyrr‐APS‐Ti_3_C_2_T*_x_* and as 3D Pt/Pyrr‐APS‐Ti_3_C_2_T*_x_*, see Experimental)—revealing the same benefits for the 3D integrated design over the powder electrode (Figure S15, Supporting Information). In all cases, however, the mass activity of the Pt catalysts in alkaline media is lower than that of the Pd‐loaded systems (Figure S15, Supporting Information), therefore, we concentrated all further performance studies on the latter.

Regarding long‐term cycling of the Pd‐based powder catalysts, Pd/Pyrr‐APS‐Ti_3_C_2_T*_x_* exhibited the best performance, as after 2000 cycles the oxidation current density thereof dropped from *≈*705 to 221 mA mg^−1^ (31.3% current density retention), while the other three powder catalyst electrodes (Pd/Pic‐APS‐Ti_3_C_2_T*_x_*, Pd/APS‐Ti_3_C_2_T*_x_* and Pd/C) rapidly lost activity after just a few hundred cycles (Figure S16, Supporting Information). All four of the 3D integrated electrodes showed better cycling stability than the corresponding powder electrodes; among them, 3D Pd/Pyrr‐APS‐Ti_3_C_2_T*_x_* showed the best electrocatalytic stability, with the current density only dropping from 842.0 to 736.9 mA mg^−1^ after 2000 cycles, i.e., exhibiting 87.5% capacity retention (Figure 4c).

Such a notable difference in the electrochemical mass activities and cycling stabilities warranted further investigation using electrochemical impedance spectroscopy (EIS) and Tafel tests. From these, it was shown that all three 3D integrated Pd‐loaded electrodes possessed charge transfer resistances at the solution/electrolyte interfaces which were about an order of magnitude lower than those of the corresponding powder electrodes (Figure 4d; Figure S17 and Table S5, Supporting Information). The 3D Pd/Pyrr‐APS‐Ti_3_C_2_T*_x_* exhibited the lowest resistance (3.5 Ω). Furthermore, as shown in Figure 4e and Figure S18 (Supporting Information), the Tafel slopes in the low polarization region for the four 3D integrated electrodes were lower than that of the corresponding four powder electrodes. Notably, 3D Pd/Pyrr‐APS‐Ti_3_C_2_T*_x_* exhibited a lower Tafel slope for the EOR (≈97.6 mV dec^−1^) than either 3D Pd/Pic‐APS‐Ti_3_C_2_T*_x_* (≈113 mV dec^−1^) or commercial Pd/C catalyst assembled in an integrated 3D Pd/C electrode (249 mV dec^−1^).

Subsequently, chronoamperometry was performed to assess the electrochemical durability of the catalysts at 0.2 V over 3600 s. The cause of the initial sharp drop in current density and subsequent trend seen in Figure S19 (Supporting Information) might be the generation and build‐up of catalyst poisons (*e.g*., CO_(ads)_ or CH_3_CO_(ads)_) on the nanoparticle surface. Expectedly, all the 3D integrated electrodes exhibited greater durability compared to the respective powder electrodes; this can be ascribed to the rich porous structure of the 3D electrodes, which favors the build‐up of oxidation intermediates without, however, affecting the cyclic voltammetric stability. Figure 4f presents prolonged cycling trials of 3D Pd/Pyrr‐APS‐Ti_3_C_2_T*_x_* and 3D Pd/Pic‐APS‐Ti_3_C_2_T*_x_* in order to assess their long‐term durability toward EOR at a scan rate of 100 mV s^−1^. 3D Pd/Pic‐APS‐Ti_3_C_2_T*_x_* showed a sustained and rapid decline in power density from ≈760 to ≈221 mA mg^−1^ with only 31.3% capacity retained after 2000 cycles. Concurrently, the multi‐layered structure of Ti_3_C_2_T*_x_* in powder Pd/Pic‐APS‐Ti_3_C_2_T*_x_* and 3D Pd/Pic‐APS‐Ti_3_C_2_T*_x_* partially collapsed after the cycling (Figure S20, Supporting Information). In contrast, 3D Pd/Pyrr‐APS‐Ti_3_C_2_T*_x_* demonstrated superior stability with up to 97.9% capacity retention and a high current density of 836.54 mA mg^−1^ after 10 000 cycles for EOR. Notably, Pd/Pyrr‐APS‐Ti_3_C_2_T*_x_* undergoes a surface reconstruction or electrochemical conditioning phase during the initial cycles. This process can lead to the exposure of a greater number of active sites, improved wetting of the electrode surface, and/or the removal of surface impurities or passivating layers. During the prolonged stability test, the formation and subsequent detachment of CO_2_ gas bubbles on the catalyst electrode surface were observed. The agitation and eventual rupture of these bubbles adsorbed onto the stable 3D electrode framework surface is hypothesised to lead to a progressive exposure of active sites, effectively increasing the electrochemically active surface area over time and contributing to the rise in current density. In addition to the improved performance, the morphology of 3D Pd/Pyrr‐APS‐Ti_3_C_2_T*_x_* after 10000 cycles (Figure S21, Supporting Information, *cf*. powder Pd/Pyrr‐APS‐Ti_3_C_2_T*_x_* after 2000 cycles in Figure S22, Supporting Information) remained essentially unchanged compared to the initial morphology (insert of Scheme 1b). Such superior stability can be related to a synergy of stronger Pd···support interactions and the 3D porous structure of the integrated electrode, providing the necessary ion channels for redox reactions within the solid 3D framework. Comparing the above data on capacity retention after extensive cycling with systems reported in the literature,^[^ ^29^, ^30^, ^31^, ^32^, ^33^, ^34^, ^35^, ^36^, ^37^, ^38^, ^39^, ^40^ ^]^ 3D Pd/Pyrr‐APS‐Ti_3_C_2_T*_x_* stands out as one among the most stable and active anodic ethanol oxidation catalysts so far created (Figure 4g; Table S6, Supporting Information), and is therefore a good candidate anodic catalyst electrode for ethanol fuel cells.

### 2.4 Mechanistic and Computational Investigations

**Figure 5:**
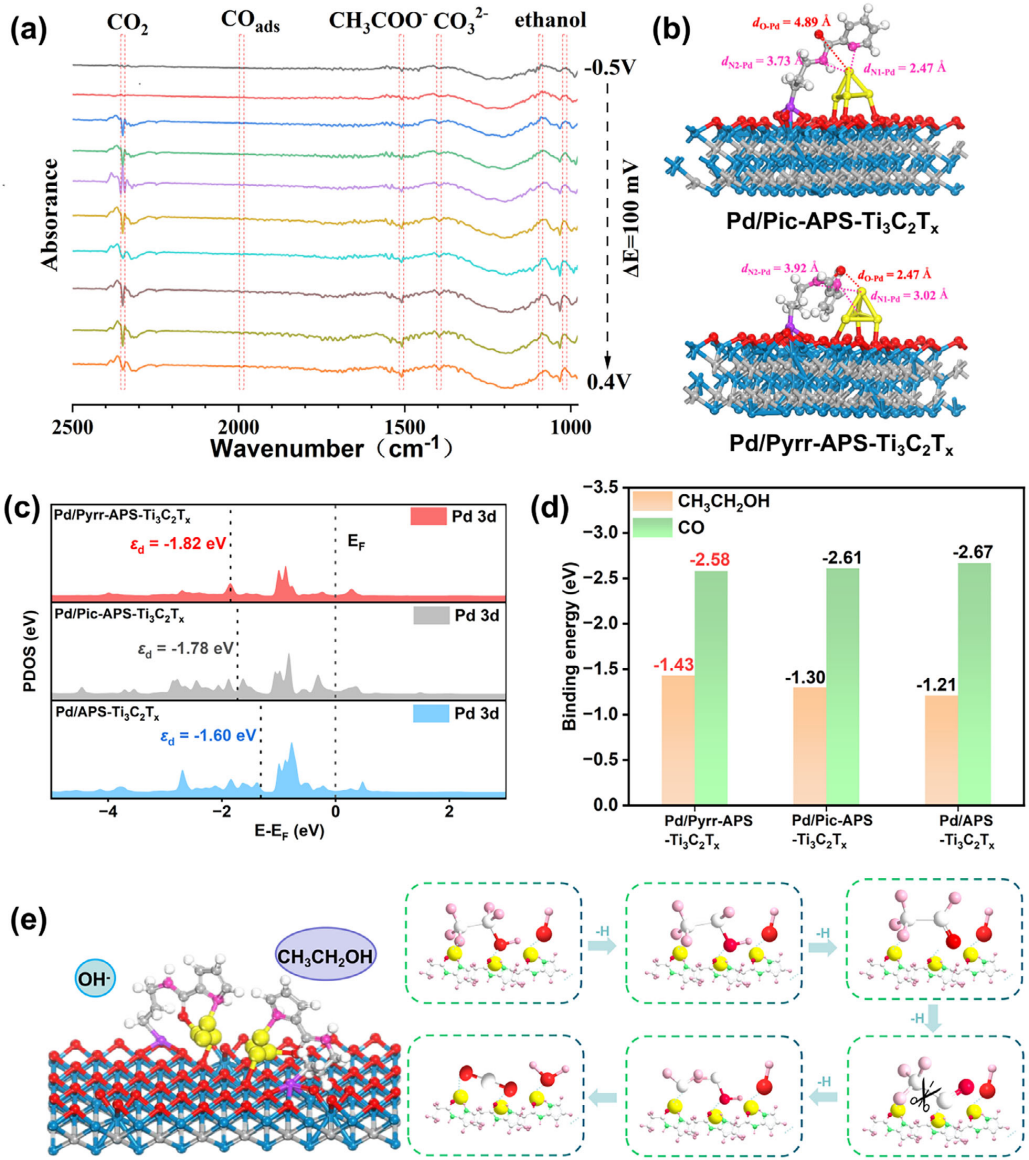
a) In situ Fourier‐transform infrared spectra (FTIR) of 3D Pd/Pyrr‐APS‐Ti_3_C_2_T*_x_* at different voltages; b) Structures obtained by ab initio molecular dynamics (AIMD) for the pyridine‐ and pyrrole‐based Pd catalysts, showing the conformation of the N‐donor around the Pd; c) Pd 3d PDOS schematic; d) Comparison of ethanol and CO adsorption energy for Pd/Pyrr‐APS‐Ti_3_C_2_T*_x_*, Pd/Pic‐APS‐Ti_3_C_2_T*_x_* and Pd/APS‐Ti_3_C_2_T*_x_*; e) Mechanistic schematic for ethanol oxidation on Pd/Pyrr‐APS‐Ti_3_C_2_T*_x_*.

To understand the intrinsic mechanism responsible for the enhanced electrocatalytic properties of the 3D Pd/Pyrr‐APS‐Ti_3_C_2_T*_x_* system, the intermediate products during the EOR process were detected with in situ FTIR (**Figure** **5**a). The characteristic bands seen in the spectrum at 1020.2 and 1081.9 cm^−1^ are assigned to C─O stretching in ethanol. The very weak peaks at 1402 and 1512 cm^−1^ belong to the symmetric and asymmetric stretching bands of O–C–O in the carbonate (CO_3_^2−^) and acetate (CH_3_COO^−^) ions, respectively.^[^ ^26^ ^]^ In addition, a band at 2351 cm^−1^ appeared, which grew gradually with increasing potential; this corresponds to the asymmetric O–C–O stretch of CO_2_, indicating high C1 selectivity (i.e., complete oxidation of ethanol) for 3D Pd/Pyrr‐APS‐Ti_3_C_2_T*_x_*. The band at 1988 cm^−1^ (assigned to the CO_(ads)_ catalyst‐poisoning intermediate) remained faint throughout the redox reaction, suggesting a very low absorption energy of the oxidation intermediates on 3D Pd/Pyrr‐APS‐Ti_3_C_2_T*_x_*.

In order to examine the mechanism further, ab initio and density functional theory (DFT) calculations were performed to construct electrocatalyst models consistent with the XPS and XAS data.^[^ ^30^ ^]^ Initially, ab initio molecular dynamics (AIMD) simulations were used to explore the exact geometry of Pd coordination in Pd/Pic‐APS‐Ti_3_C_2_T*_x_* and Pd/Pyrr‐APS‐Ti_3_C_2_T*_x_*; the results are shown in Figure 5b. The two catalysts exhibited (as expected on the basis of the prior Raman spectroscopic characterisation) different Pd—ligand interactions: for Pd/Pic‐APS‐Ti_3_C_2_T*_x_*, a short distance was obtained between Pd and the pyridine N (*d*_N1‒Pd_: 2.47 Å), and large distances to the amidic N (*d*_N2‒Pd_: 3.73 Å) and O (*d*_O‒Pd_: 4.89 Å), indicating that the Pd atoms are bound strongly to the pyridine moiety and essentially lack any potential interaction with the amide functionality of the linker. In contrast, for Pd/Pyrr‐APS‐Ti_3_C_2_T*_x_*, the Pd atoms were calculated to be close to both the pyrrolic N (*d*_N1‒Pd_: 3.02 Å) and amidic O (*d*_O‒Pd_: 2.47 Å), but distant from the amidic N (*d*_N2‒Pd_: 3.92 Å), hinting at the possibility of utilising the amide and pyrrole moieties to co‐anchor Pd in a κ^2^‐(*O*,*N*) bidentate manner. This difference in behaviour is unsurprising given the very different electron‐donating abilities of the two ligands: pyridines typically make very stable transition metal complexes (even as monodentates), whereas the behaviour of the pyrrole is consistent with the ring orientation observed in the only crystallographically characterised transition metal complex of a neutral pyrrolic ring (where it is part of a tridentate κ^2^‐(*P,N,N*) pincer ligand binding ruthenium).^[^ ^39^ ^]^ In addition, the adsorption energies of Pd and other N linkers (*e.g*., pyrrole, amide) help quantify the interaction of Pd/Pyrr‐APS‐Ti_3_C_2_T*_x_* (Figure S23, Supporting Information). The palladium adsorption energy of the co‐interacting amidic O and pyrrolic N donors was the strongest among these Pd‐containing structures at −5.61 eV (*cf*. −5.24 eV for Pd/Pic‐APS‐Ti_3_C_2_T*_x_* and −4.87 eV for Pd/APS‐Ti_3_C_2_T*_x_*), representing the most increased structural stability of Pd/Pyrr‐APS‐Ti_3_C_2_T*_x_* owing to this “hand‐in‐hand” co‐anchoring interaction (Figure S24, Supporting Information).

A corresponding variation in electrocatalytic performance for the different ligand‐functionalised materials is also expected, as the drastic effects on catalytic properties upon changing the heterocyclic donors in transition metal complexes have been explored extensively in the literature.^[^ ^41^ ^]^ In particular, with respect to this system, differential charge density calculations (Figure S25, Supporting Information) reveal that the Pd atoms in Pd/Pyrr‐APS‐Ti_3_C_2_T*_x_* are more partially negatively charged (+0.94 e^−^) than in Pd/Pic‐APS‐Ti_3_C_2_T*_x_* (+0.88 e^−^) and Pd/APS‐Ti_3_C_2_T*_x_* (+0.87 e^−^), thereby facilitating the desorption of adsorbed intermediates. The d‐band centre for the Pd atoms adsorbing CO in Pd/Pyrr‐APS‐Ti_3_C_2_T*_x_* is located at −1.82 eV in the modelled DOS (Figure 5c), which is lower than that in Pd/Pic‐APS‐Ti_3_C_2_T*_x_* (‐1.78 eV) and Pd/APS‐Ti_3_C_2_T*_x_* (−1.60 eV); this supports the conclusion that there is diminished catalyst poisoning from oxidation intermediates (e.g., CO_(ads)_) in the best‐performing system. Three representative atomic models for the adsorption of ethanol and CO on the Pd···N site of the functionalised Ti_3_C_2_T*_x_* nanosheets are shown in Figure S26 (Supporting Information) and Figure 5d. The ethanol adsorption energy for Pd/Pyrr‐APS‐Ti_3_C_2_T*_x_* was calculated at −1.43 eV, which is greater than that for Pd/Pic‐APS‐Ti_3_C_2_T*_x_* (−1.30 eV) and Pd/APS‐Ti_3_C_2_T*_x_* (−1.21 eV), indicating comparably stronger adsorption of ethanol on the pyrrole‐functionalised MXene catalyst. Similarly, the CO adsorption energy for Pd/Pyrr‐APS‐Ti_3_C_2_T*_x_* was calculated to be −2.58 eV, higher than that for Pd/Pic‐APS‐Ti_3_C_2_T*_x_* (−2.61 eV) and Pd/APS‐Ti_3_C_2_T*_x_* (−2.67 eV), providing further evidence of the weakened interaction between CO_(ads)_ and the pyrrole‐based catalyst. Overall, the above theoretical calculations are in agreement with the experimentally observed ranking of the electrocatalytic performance of the three systems; they show how the modified surface of the Pd/Pyrr‐APS‐Ti_3_C_2_T*_x_* catalyst can affect the Pd atoms in such a way that the adsorption of ethanol and OH^·^ are enhanced whereas that of CO is disfavoured (Figure 5e), aiding the cleavage of C‒C bonds and thereby facilitating EOR by a favourable C1 pathway.^[^ ^42^ ^]^

## 3 Conclusion

In summary, we have presented a new approach on the design of integrated electrodes for Pd‐catalysed ethanol oxidation, consisting of a layered 3D “sandwich”‐like structure in which the Pd nanoparticles have atomically well‐defined active sites thanks to chemical functionalisation of the MXene support. The strong interactions of Pd nanoparticles with the support are evident from the significantly improved average Pd‒Pd and Pd‒N/O coordination numbers, and also the catalyst's unique morphology (with uniform Pd coverage and directional exposure of the Pd (111) and (222) crystal surfaces). One of the as‐assembled novel 3D electrodes (3D Pd/Pyrr‐APS‐Ti_3_C_2_T*_x_*) stands out for being highly cyclable for up to 10 000 cycles with a capacity retention of 97.9%, and is one of the most stable and efficient electrocatalytic systems reported for this reaction. In situ FTIR and computational modelling hint at a low absorption energy for the oxidation intermediates, thereby facilitating EOR through a favourable C1 pathway; in combination with the preservation of the material's layered morphology upon cycling, these results highlight the pyrrole‐functionalised 3D integrated electrode as a particularly promising system. The presence of well‐defined catalytic sites provides opportunities for in‐depth investigation of the catalytic mechanism in systems designed with this approach—our prototype provides an important basis for the systematic modulation of functional electrodes in this area, aiming to achieve improved performance for real‐world applications in fuel cells.

## Conflict of Interest

The authors declare no conflict of interest.

## Supporting information



Supporting Information

## Data Availability

The data that support the findings of this study are available from the corresponding author upon reasonable request.
