# Supplementary material for: Sustained and Efficient Ethanol Oxidation by Tethering Tunable N‐Donors Onto Integrated MXene/Pd‐Electrodes
Source: Adv Sci (Weinh). 2025 Oct 13;13(2):e13512. doi: 10.1002/advs.202513512 (PMC12786351; doi:10.1002/advs.202513512)
Supplement: Supplementary file 1 — Supporting Information [file ADVS-13-e13512-s001.docx]

Supporting Information

Sustained and Efficient Ethanol Oxidation by Tethering Tunable N-donors onto Integrated MXene/Pd-electrodes

*Zhangxin Chen* ^‡^*^, a, b, c, d^, Fan Jing* ^‡^*^, c^, Alexandros Terzopoulos* ^‡^*^, b^, Yanxian Jin*^, a, c^, Kai Huang ^c,e^, Haichang Fu ^*, a, c, d^, Na Liu ^c^, Xianqiang Xiong ^a, c^, Binbin Yu ^a, c^, Dan Chen ^a, c^, Yang Xia ^f^, Dominic S. Wright ^*, b^*

^a^ Zhejiang Key Laboratory for Island Green Energy and New Materials, Taizhou University, Taizhou 318000, Zhejiang, China

^b^ Yusuf Hamied Department of Chemistry, University of Cambridge, Lensfield Road, Cambridge CB2 1EW, UK

^c^ School of Pharmaceutical and Chemical Engineering, Taizhou University, Taizhou 318000, Zhejiang, China

^d^ Taizhou Biomedical and Chemistry Industry Institute, Taizhou 318000, Zhejiang, China

^e^ School of Chemistry and Molecular Engineering, East China University of Science and Technology, Shanghai 200237, Shanghai, China

^f^ College of Materials Science and Engineering, Zhejiang University of Technology, Hangzhou 310014, Zhejiang, China

^‡^ These authors contributed equally to this work; all three authors should be considered first authors of the paper.

1. Experimental Section

1.1. Materials

Ti_3_AlC_2_ (500 mesh, >98 wt%) was purchased from Laizhou Kai Ceramic Materials Co. (3-Aminopropyl)trimethoxysilane (APTMS), pyrrole-2-carboxylic acid, picolinic acid, 4‑dimethylaminopyridine (DMAP), *N,N*-dicyclohexylcarbodiimide (DCC), toluene, glacial acetic acid, absolute ethanol (>99.5 wt%), single-walled carbon nanotubes (SWCNT, >99.5 wt%), polyvinylpyrrolidone (PVP), Pd/C (20 wt% Pd), dichloromethane (>99.5wt%), potassium borohydride and palladium(II) chloride were obtained from Adamas Reagent Ltd. Concentrated hydrofluoric acid (>40 wt% HF), ammonium hydroxide aqueous solution (25‒28 wt% NH_3_) were obtained from Shanghai Chemical Reagents Co. All the reagents, unless otherwise specified, were of analytical grade (>99 wt%) and used without further purification.

1.2. Sample preparation

1.2.1 Preparation of Ti_3_C_2_T_x_

Following the established experimental procedure, Ti_3_C_2_T*_x_* was obtained by etching the Ti_3_AlC_2_ ceramic precursor with HF solution: 2 g of Ti_3_AlC_2_ were homogeneously dispersed in 10 mL of 40 wt% HF solution and stirred at room temperature for 28 h. Subsequently, the mixture was centrifuged at 10000 rev·min^-1^ for 10 min each time, and washed repeatedly with deionised water several times until the pH value of the washing solution was close to 6. Following the centrifugation-induced deaggregation, the prepared Ti_3_C_2_T*_x_* nanosheets were dried in a vacuum oven at 80 ^o^C overnight to obtain a black powder of *ca* 85 % yield (typical product mass: 1.46 g).

1.2.2 Preparation of APS-Ti_3_C_2_T_x_

200 mg of Ti_3_C_2_T*_X_* nanosheets prepared as above were dissolved in a mixture of deionised water (67 mL) and absolute ethanol (133 mL) (Solution A). 830 mg of APTMS were dissolved in absolute ethanol (10 mL) (Solution B). Solution A and solution B were mixed, the pH was adjusted to *ca* 3.5 with dropwise addition of acetic acid, and the resulting mixture then stirred at room temperature for 24 h under a N_2_ atmosphere. Finally, this was filtered, washed with ethanol, and dried *in vacuo* at 80 ^o^C overnight to afford the aminosilane-functionalised MXene (APS-Ti_3_C_2_T*_x_*) as a grey powder (320 mg yield).

1.2.3 Preparation of Pyrr-APS-Ti_3_C_2_T_x_ and Pic-APS-Ti_3_C_2_T_x_

APS-Ti_3_C_2_T*_x_* (80 mg) was dissolved in 50 mL of dichloromethane. To the solution were added sequentially pyrrole-2-carboxylic acid (220 mg), DCC (620 mg) and DMAP (20 mg). The mixture was stirred under a N_2_ atmosphere at room temperature for 3 days. Finally, it was isolated *via* filtration, washed with deionised water for three times, and dried *in vacuo* at 80 ^o^C for 12 h to obtain Pyrr-APS-Ti_3_C_2_T*_x_* as a grey powder (110 mg yield). The picolinic acid-modified MXene support (Pic-APS-Ti_3_C_2_T*_x_*) was synthesized in a 100 mg yield using a similar method starting from APS-Ti_3_C_2_T*_x_* (80 mg) and picolinic acid (220 mg).

1.2.4 Pd and Pt loading on the support

Pd/Pyrr-APS-Ti_3_C_2_T*_x_*, Pd/Pic-APS-Ti_3_C_2_T*_x_* and Pd/APS-Ti_3_C_2_T*_x_* were prepared by reduction of palladium(II) chloride with potassium borohydride in a suspension of the respective functionalised MXene support. Taking Pd/Pyrr-APS-Ti_3_C_2_T*_x_* as an example, the prepared Pyrr-APS-Ti_3_C_2_T*_x_* (80 mg) was dispersed ultrasonically in deionised water (30 mL) for 15 min; thereto was added an aqueous solution of PdCl_2_ (0.05 m, 3.76 mL) and the pH was adjusted to 7 by dropwise addition of dilute ammonium hydroxide solution. The sample was heated first at 50 ^o^C for 1 h, then an aqueous solution of KBH_4_ (0.06 m, 160 mL) was added dropwise over 3 h and let stir for a further 3 h at 50 ^o^C. The final product (Pd/Pyrr-APS-Ti_3_C_2_T*_x_*) was filtered, washed and dried *in vacuo* at 70 ^o^C for 12 h, resulting a black powder (68 mg yield). The Pd/Pic-APS-Ti_3_C_2_T*_x_* and Pd/APS-Ti_3_C_2_T*_x_* catalysts were prepared in similar yields using an analogous method. A platinum-loaded homologue of the pyrrole-based catalyst (Pt/Pyrr-APS-Ti_3_C_2_T*_x_*) was prepared in an analogous method and yield, using potassium hexachloroplatinate(IV) (K_2_PtCl_6_) as the metal precursor for reductive deposition.

1.2.5 Preparation of integrated-electrode 3D materials

The active and support layers were assembled in a sandwich-like fashion (see **Scheme 1b**). Taking 3D Pd/Pyrr-APS-Ti_3_C_2_T*_x_* as an example, for the preparation of the active layer (AL), Pd/Pyrr-APS-Ti_3_C_2_T*_x_* (50 mg), SWCNT (50 mg) and PVP (50 mg) were dispersed in toluene (100 mL) and stirred for 10 h at room temperature until a uniformly dispersed suspension formed. For the preparation of the support layer (SL), SWCNT (50 mg) and Ti_3_C_2_T*_x_* (150 mg) were dispersed in toluene (100 mL) and stirred at room temperature for 10 h until a uniformly dispersed suspension formed. The assembly into a “sandwich” structure was achieved through layer-by-layer *vacuo* filtration, with a loading of about 0.8~1 mg cm^‒2^ in each iteration: on the first filter were deposited 20 ml of the support layer suspension to form the lower SL, followed by 20 ml of the active layer suspension to form the intermediate AL, and finally 20 ml of the support layer suspension to form the upper SL. The 3D Pd/Pic-APS-Ti_3_C_2_T*_x_*, 3D Pt/Pyrr-APS-Ti_3_C_2_T*_x_*, 3D Pd/APS-Ti_3_C_2_T*_x_* and 3D Pd/C were prepared using an analogous method.

1.3 Materials characterisation

X-ray photoelectron spectra (XPS) were measured using a Thermo Scientific K-Alpha instrument. Raman spectroscopic measurements were performed on a HORIBA JY LabRAM HR Evolution confocal Raman microscope at 532 nm. Powder X-ray diffraction (PXRD) experiments were undertaken on a Philips PW3040/60 X-ray diffractometer using Cu Kα radiation (*λ* = 0.1541 nm). The patterns were collected in a 2*θ* range from 5° to 80° with a step of 0.0167°. For scanning electron microscopy (SEM), a Hitachi S-4800 device was used. Transmission electron microscopy (TEM) and mapping experiments were performed on a FEI Tecnai G2 F30 TEM with accelerating voltage of 300 kV. The National Synchrotron Radiation Research Center in Taiwan 44A beamline was used to gather the V K-edge X-ray absorption fine structure (XAFS) spectra. The data were collected in transmission mode using a Lytle detector.

1.4 Electrochemical measurements

Electrochemical measurements were carried out using a CHI760 electrochemical workstation in a conventional three-electrode cell with one of the prepared integrated electrodes (*e.g.* 3D Pd/Pyrr-APS-Ti_3_C_2_T*_x_*) as the working electrode, Pt wire as the counter electrode and a Hg/HgO electrode as the reference electrode. The catalyst dosage was determined using inductively coupled plasma optical emission spectrometry (ICP-OES), the results of which can be found in the Supporting Information. All the tests were performed under an inert atmosphere and at a temperature of 25°C. The *E*_RHE_ can be obtained by the equation: *E*_RHE_ = *E*_test_ + *E*^o^_Hg/HgO_ + 0.059*pH, where *E*^o^_Hg/HgO_ =0.098 V, pH= 14.

1.5 *Ab initio* and DFT calculations

All calculations were executed using the Vienna *Ab Initio* Simulation Package (VASP) with a projector augmented ware (PAW) pseudopotential. The exchange and correlation energies were established by the generalized gradient approximation (GGA) with a Perdew-Burke-Ernzerhof functional (PBE), and weak interactions were accounted of with the DFT-D3 dispersion-corrected method.^[1]^ The Gaussian electron smearing *σ* was set to 0.05 eV and the energy cut-off was set to 400 eV. All atoms in the structure are relaxed until the force and energy were less than 0.02 eV Å^‒1^ and 10^‒4^ eV/atom, respectively. All the models were constructed in a 15.3 Å × 15.3 Å × 24.7 Å hexagonal box, and a 20 Å vacuum layer was adopted in the *z*-direction to avoid periodic interactions. A 3 × 3 × 1 Monkhorst-Pack K-point grid was constructed for all models. The energies of CO_(ads)_ and CH_3_CH_2_OH_(ads)_ were calculated in a 15.0 Å × 15.0 Å × 15.0 Å cubic box with a 1 × 1 × 1 Gamma K-point grid. The binding energy (*E*_b_) was obtained by *E*_b_ = *E*_t_ - *E*_slab_ - *E*_M_, where *E*_t_ is the total energy of the system, *E*_slab_ is the energy of the MXene slab with a Pd cluster, and *E*_M_ is the energy of the adsorbed molecule (CO or CH_3_CH_2_OH). *Ab initio* molecular dynamics (AIMD) simulations were carried out employing a 1 × 1 × 1 Gamma-point grid for Brillouin zone sampling. The simulations were conducted in the NVT ensemble, using a Nosé-Hoover thermostat to maintain a constant temperature of 600 K. The total trajectory length for the AIMD simulation was 1.5 ps.

**References for Experimental**

1. S. Grimme, J. Antony, S. Ehrlich, H. Krieg. *J. Chem. Phys.* **2010**, 132, 154104.

Table Section

**Table S1** Pd content of the eight electrode samples as determined by ICP

| Samples | wt%/ ICP | Pd mass on the  electrode (mg) ^a, b^ |
| --- | --- | --- |
| 3D Pd/Pyrr-APS-Ti_3_C_2_T_x_ | 9.1% | 0.0322 |
| 3D Pd/Pic-APS-Ti_3_C_2_T_x_ | 19.7% | 0.0697 |
| 3D Pd/APS-Ti_3_C_2_T_x_ | 15.6% | 0.0552 |
| 3D Pd/C | 20% | 0.0708 |
| Powder Pd/Pyrr-APS-Ti_3_C_2_T_x_ | 9.1% | 0.0493 |
| Powder Pd/Pic-APS-Ti_3_C_2_T_x_ | 19.7% | 0.1067 |
| Powder Pd/APS-Ti_3_C_2_T_x_ | 15.6% | 0.0844 |
| Powder Pd/C | 20% | 0.1083 |

^a^ The 3D Pd mass of catalyst electrode: *m* = 10 mg * 0.36 cm^2^/10.17 cm^2^ * wt%,

^b^ The powder Pd mass of catalyst electrode: *m* = 5 mg * 3.25 μL/30 μL * wt%

**Table S2** Elemental contents of the active catalysts as determined by XPS

| Sample | Element Content/ at% | | | | | |
| --- | --- | --- | --- | --- | --- | --- |
|  | C | N | O | Si | Ti | Pd |
| Pd/Pyrr-APS-Ti_3_C_2_T_x_ | 48.11 | 11.08 | 15.33 | 1.54 | 20.44 | 3.5 |
| Pd/Pic-APS-Ti_3_C_2_T_x_ | 27.51 | 4.02 | 37.62 | 3.68 | 16.64 | 10.53 |
| Pd/APS-Ti_3_C_2_T_x_ | 37.41 | 7.53 | 23.21 | 3.27 | 21.01 | 7.57 |

**Table S3** EXAFS fitting parameters at the Pd K-edge for two catalyst samples (*S*_0_^2^=0.8508)

| Samples | path | C N^a^ | *R* (Å) ^b^ | *σ*^2^(Å^2^)^c^ | Δ*E*_0_ (eV) ^d^ | *R* factor |
| --- | --- | --- | --- | --- | --- | --- |
| Pd/Pyrr-APS-Ti_3_C_2_T_x_ | Pd-O/N | 1.6±0.1 | 2.03 | 0.006 | -6.0±0.2 | 0.002 |
|  | Pd-Pd | 7.0±0.1 | 2.74 | 0.005 |  |  |
| Pd/Pic-APS-Ti_3_C_2_T_x_ | Pd-O/N | 0.2±0.1 | 2.00 | 0.001 | 4.4±0.2 | 0.003 |
|  | Pd-Pd | 2.5±0.1 | 2.74 | 0.003 |  |  |
|  | Pd-Cl | 1.1±0.1 | 2.25 | 0.010 |  |  |

^a^CN, coordination number; ^b^*R*, the distance to the neighboring atom; ^c^*σ*^2^, the Mean Square Relative Displacement (MSRD); ^d^Δ*Ε*_0_, inner potential correction; *R* factor indicates the goodness of the fit. *S*_0_^2^ was fixed to 0.8508, according to the experimental EXAFS fit of the sample foil by fixing CN as the known crystallographic value. This value was fixed during EXAFS fitting, based on the known structure of Pd foil. Data ranges 3.0 ≤ *k* ≤ 11.0 Å^-1^, 1.0 ≤ *R* ≤ 3.0 Å. The Debye-Waller factors and Δ*R*s are based on the guessing parameters and constrained for paths.

**Table S4** Calculated ECSAs of the eight catalysts

| Sample | Pd mass on the  electrode (mg) | Fitting peak area (C) | *ECSA* (m^2^/g)^a^ |
| --- | --- | --- | --- |
| 3D Pd/Pyrr-APS-Ti_3_C_2_T_x_ | 0.0322 | 0.0229 | 175.6 |
| 3D Pd/Pic-APS-Ti_3_C_2_T_x_ | 0.0697 | 0.0105 | 37.2 |
| 3D Pd/APS-Ti_3_C_2_T_x_ | 0.0552 | 0.0076 | 34.0 |
| 3D Pd/C | 0.0708 | 0.0309 | 107.8 |
| Powder Pd/Pyrr-APS-Ti_3_C_2_T_x_ | 0.0493 | 0.0059 | 29.5 |
| Powder Pd/Pic-APS-Ti_3_C_2_T_x_ | 0.1067 | 0.0025 | 5.6 |
| Powder Pd/APS-Ti_3_C_2_T_x_ | 0.0844 | 0.0004 | 1.2 |
| Powder Pd/C | 0.1083 | 0.0115 | 26.2 |

^a^ *ECSA* = *Q*/ (*m*_Pd_ * 0.405), see main text

**Table S5** Calculated impedance of the eight electrocatalytic systems

| Samples | *R*_ct_ (Ω) |
| --- | --- |
| 3D Pd/Pyrr-APS-Ti_3_C_2_T_x_ | 3.5 |
| 3D Pd/Pic-APS-Ti_3_C_2_T_x_ | 8.3 |
| 3D Pd/APS-Ti_3_C_2_T_x_ | 11.2 |
| 3D Pd/C | 9.5 |
| Powder Pd/Pyrr-APS-Ti_3_C_2_T_x_ | 103.7 |
| Powder Pd/Pic-APS-Ti_3_C_2_T_x_ | 120.2 |
| Powder Pd/APS-Ti_3_C_2_T_x_ | 1689.4 |
| Powder Pd/C | 420.3 |

**Table S6** Collected catalytic activities and stabilities of the Pd catalysts towards EOR

| Catalyst | Electrolyte | Catalytic activity | Stability | Ref  (main text) |
| --- | --- | --- | --- | --- |
| Pd/DB-Ti_3_C_2_ | 1 M KOH + 1 M CH_3_CH_2_OH | 65.4mA/cm^2^ | 91.1% after 2000 cycles | 8 |
| Pd/PANI-MWCNTsSnO_2_/Ti | 0.5MKOH + 1 M CH_3_CH_2_OH | 0.4365mA/cm^2^ | 72.88% after 700cycles | 29 |
| Pd_0.5_-Au_1.5_/MXene | 1 M KOH + 1 M CH_3_CH_2_OH | 76.47mA/mg | 73.6% after 500cycles | 30 |
| Pd/Ti_3_C_2_T_x_@NG | 1 M KOH + 1 M CH_3_CH_2_OH | 2262.2 mA/mg | 31.45% after 500cycles | 31 |
| Au NF@Pd | 1 M KOH + 1 M CH_3_CH_2_OH | 9.8A/mg | 4.07% after 1000cycles | 32 |
| L-Pd aerogel | 1 M KOH + 1 M CH_3_CH_2_OH | 2.31A/mg | 4.07% after 300cycles | 33 |
| Pd/HTC-N1.67%CNTs | 1 M KOH + 1 M CH_3_CH_2_OH | 8.3mA/cm^2^ | 29% after 800cycles | 34 |
| Pd_76_Ag_24_WNWs | 1 M KOH + 1 M CH_3_CH_2_OH | 3.5A/mg | 66% after 250cycles | 35 |
| Au/Pd | 1 M KOH + 1 M CH_3_CH_2_OH | 0.61A/mg | 45.6% after 800cycles | 36 |
| CoP/RGO-Pd10 | 1 M KOH + 1 M CH_3_CH_2_OH | 2500 mA/mg | 78.3% after 250cycles | 37 |
| Pd/B-N-Ti_3_C_2_ | 1 M KOH + 1 M CH_3_CH_2_OH | ~74.6 mA/cm^2^ | 68.7% after 400 cycles | 10 |
| Pd/GO_5_-MXene_5_-PS | 1 M KOH + 1 M CH_3_CH_2_OH | 2944.0 mA/mg | 55.4% after 800 cycles | 38 |
| Pd/N-Ti_3_C_2_ | 1 M KOH + 1 M CH_3_CH_2_OH | ~36 mA/cm^2^ | 78.3% after 350 cycles | 9 |
| Powder Pd/Pyrr-APS-Ti_3_C_2_T_x_ | 1 M KOH + 1 M CH_3_CH_2_OH | 698.29 mA/mg or 272.42mA/cm^2^ | 31.3% after 2000 cycles | This work |
| 3D Pd/Pyrr-APS-Ti_3_C_2_T_x_ | 1 M KOH + 1 M CH_3_CH_2_OH | 931.58 mA/mg | 97.9% after 10000 cycles | This work |

**Figure Section**

**Figure S1**


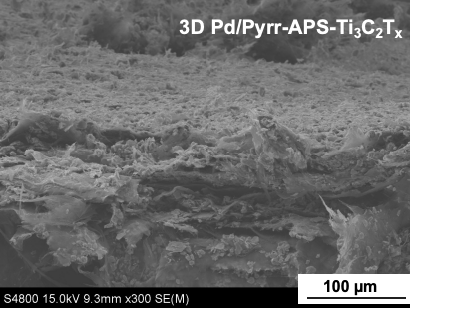


**Figure S1** SEM image of the cross-section of 3D Pd/Pyrr-APS-Ti_3_C_2_T*_x_*.

**Figure S2**


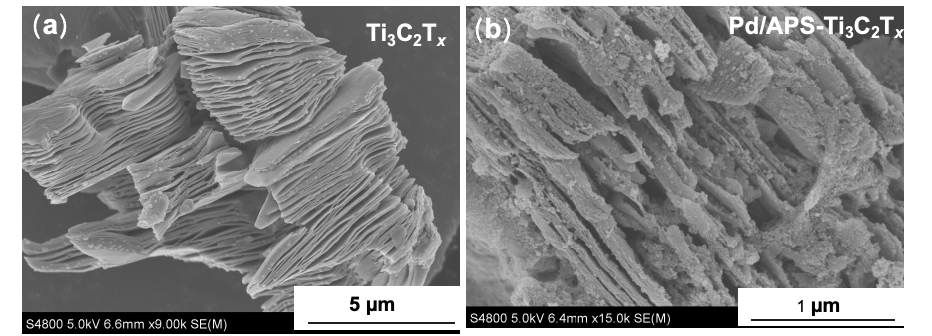


**Figure S2** SEM images of: a) unfunctionalised Ti_3_C_2_T*_x_*; b) Pd/APS-Ti_3_C_2_T*_x_*.

**Figure S3**


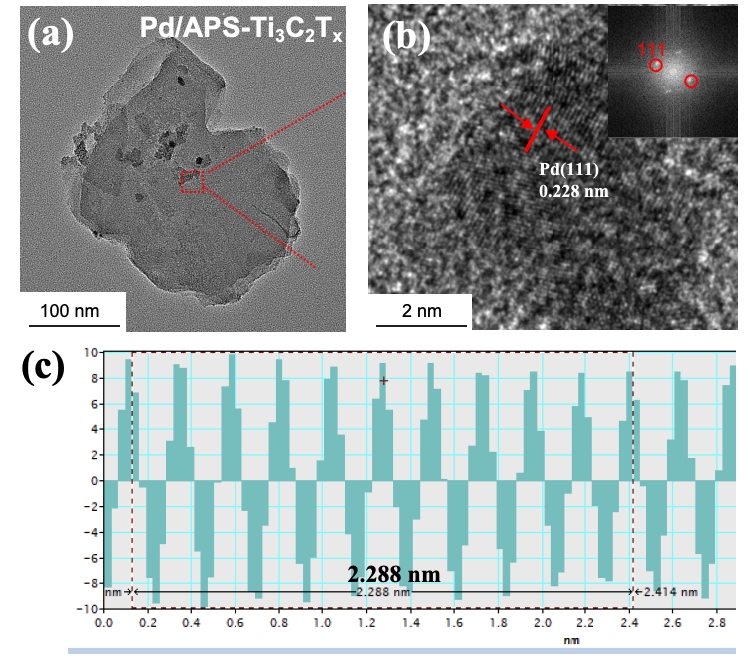


**Figure S3** TEM of Pd/APS-Ti_3_C_2_T*_x_*: a) TEM image; b) HRTEM image—insert: FFT pattern; (c) Pd (111) crystal size measurement from the HR-TEM image*.*

**Figure S4**


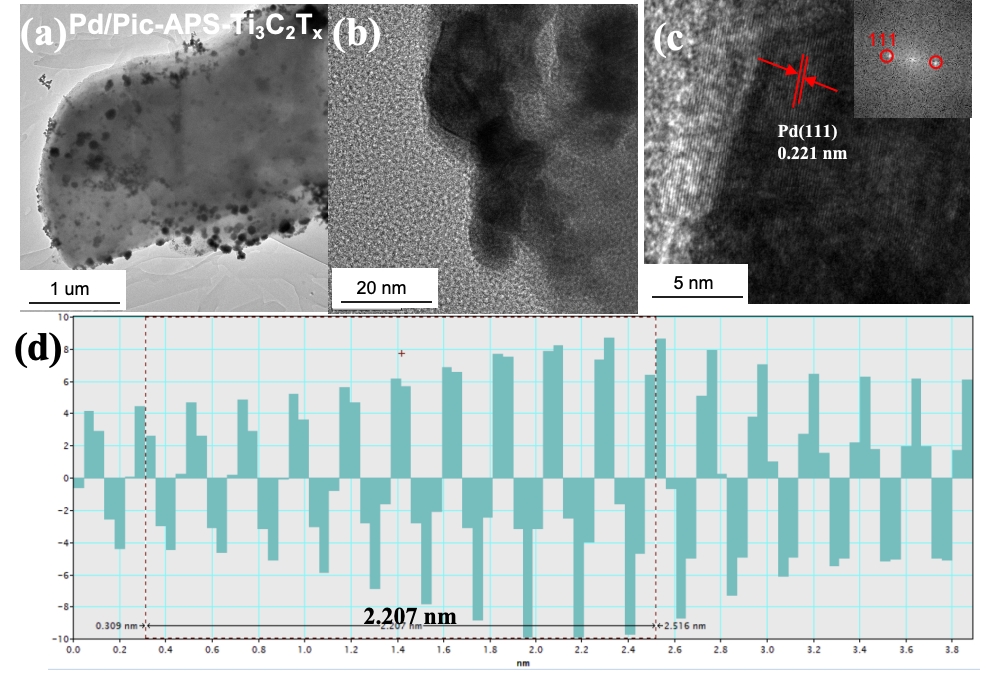


**Figure S4** TEM of Pd/Pic-APS-Ti_3_C_2_T*_x_*: a) TEM images; b) zoomed-in section; c) HR-TEM image—insert: FFT pattern; (c) Pd (111) crystal size measurement from HR-TEM.

**Figure S5**


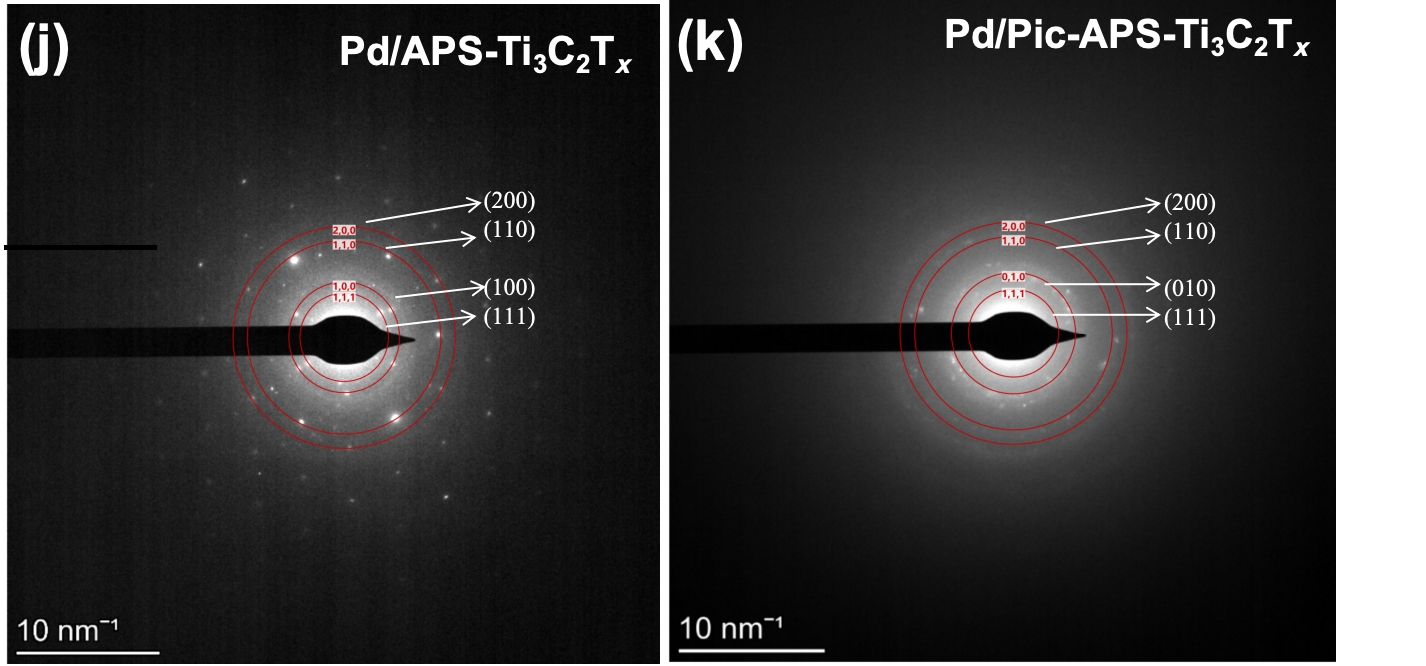


**Figure S5** SAED patterns of Pd/APS-Ti_3_C_2_T*_x_* and Pd/Pic-APS-Ti_3_C_2_T*_x_*.

**Figure S6**

**_
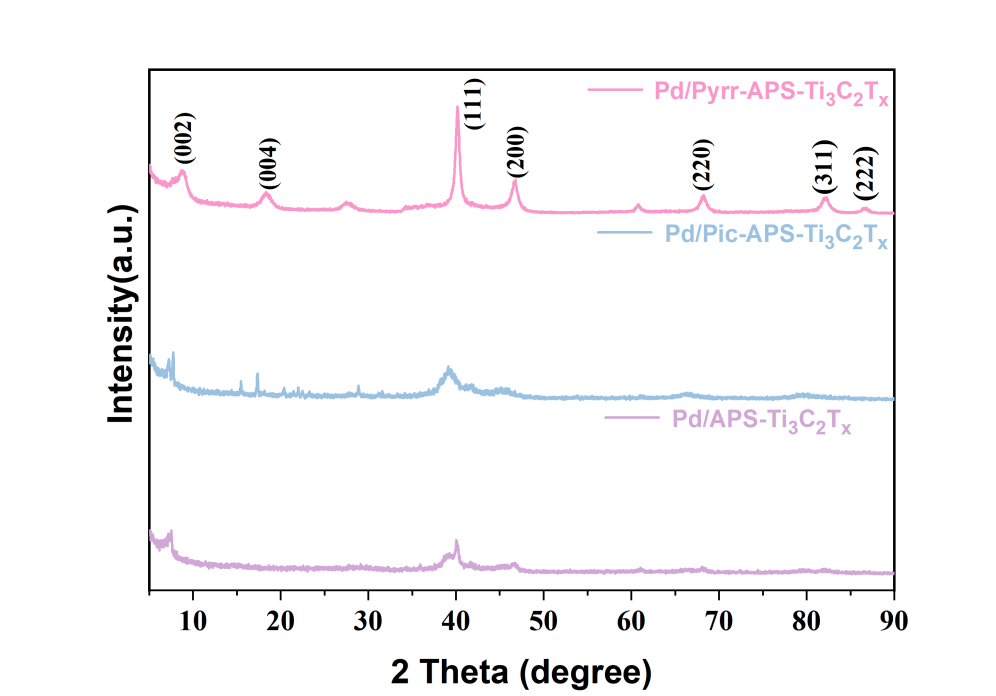
_**

**Figure S6** PXRD patterns of Pd/Pyrr-APS-Ti_3_C_2_T*_x_*_,_Pd/Pic-APS-Ti_3_C_2_T*_x_* and Pd/APS-Ti_3_C_2_T*_x_*_._

**Figure S7**


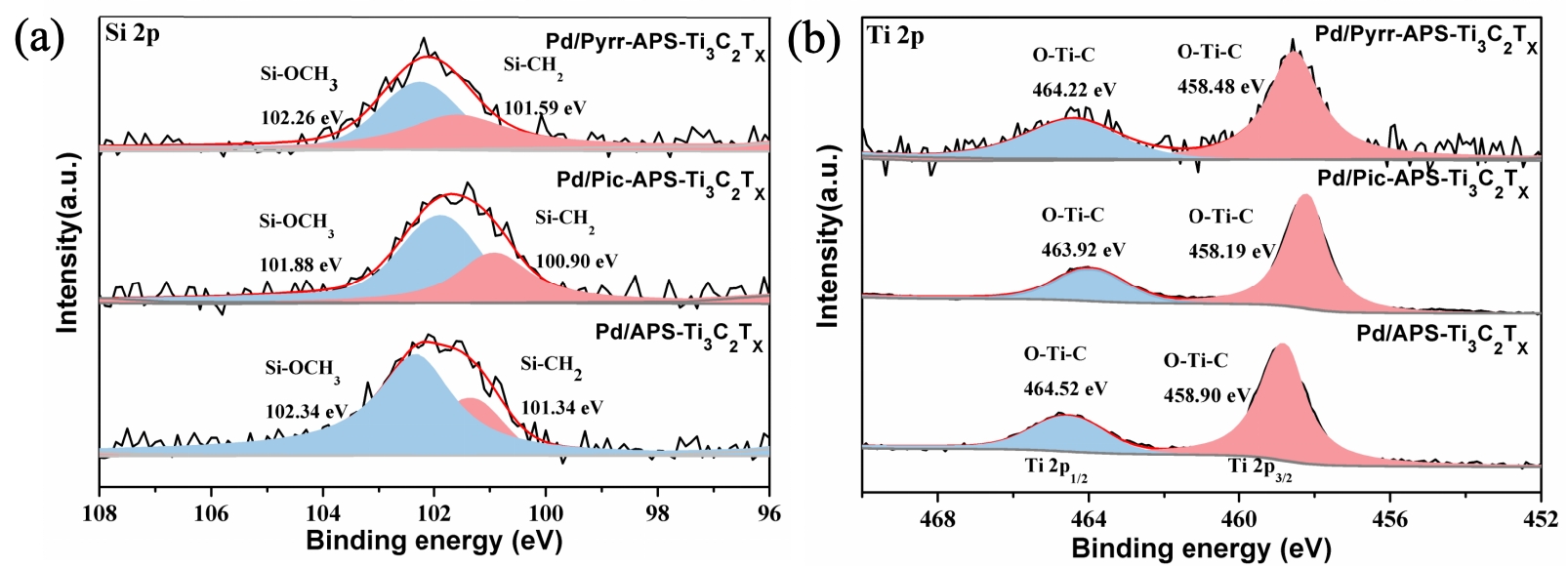
**Figure S7**  XPS element fitting for: a) Si 2p; b)Ti 2p_1/2_ and 2p_3/2_ of Pd/Pyrr-APS-Ti_3_C_2_T*_x_*_,_ Pd/Pic-APS-Ti_3_C_2_T*_x_* and Pd/APS-Ti_3_C_2_T*_x_* _._

**Figure S8**


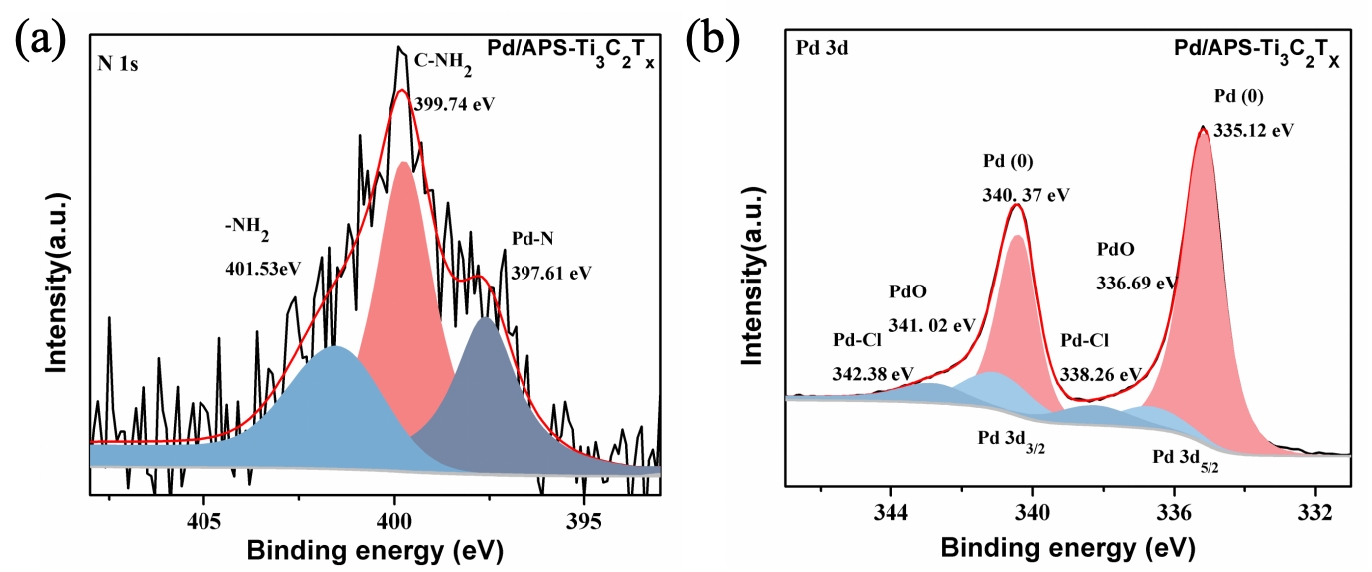


**Figure S8** XPS elemental fitting for: a) N 1s; b) Pd 3d of Pd/APS-Ti_3_C_2_T*_x_* _._

**Figure S9**

_
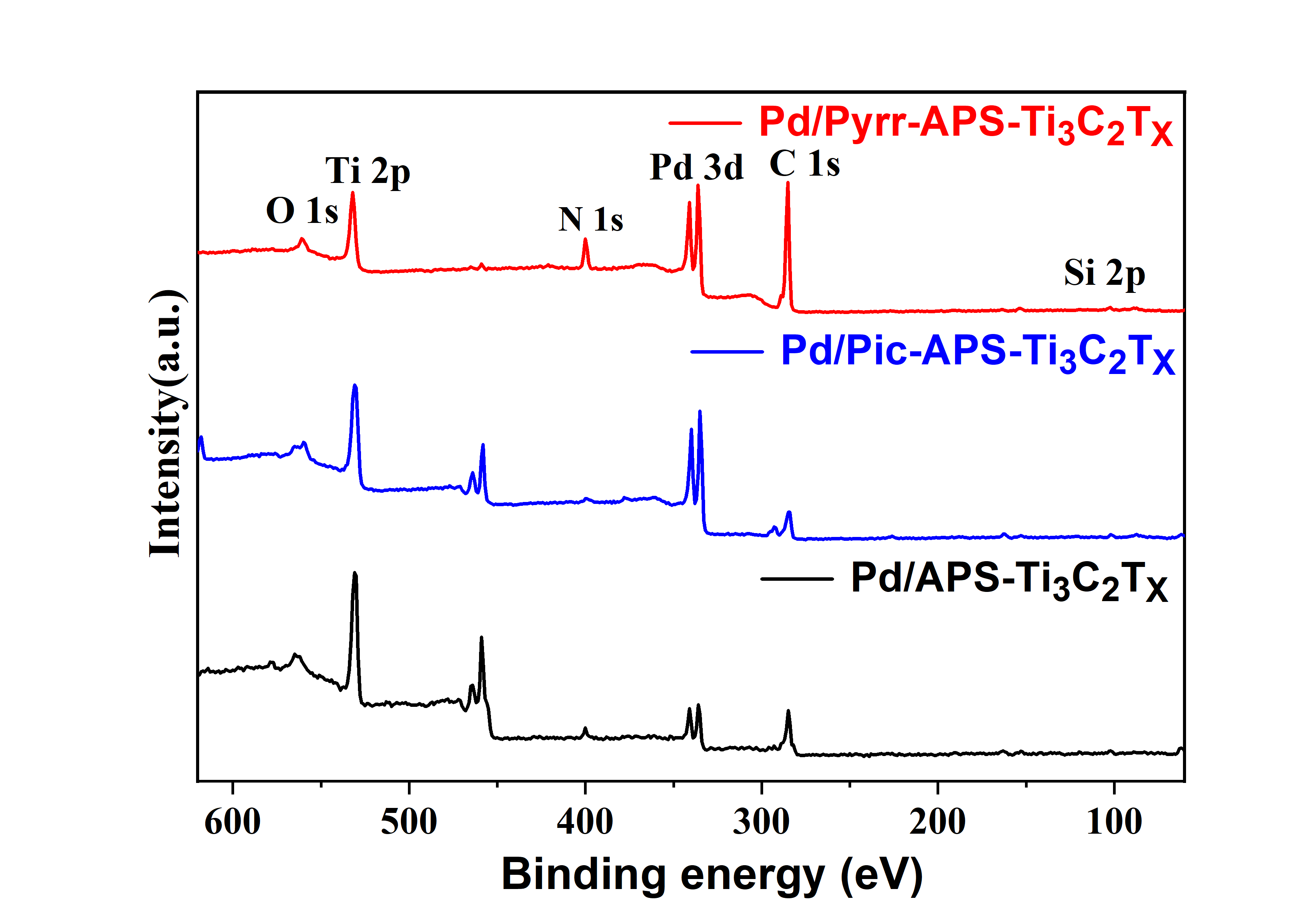
_

**Figure S9** XPS broad spectra of Pd/Pyrr‑APS‑Ti_3_C_2_T*_x_*_,_ Pd/Pic-APS-Ti_3_C_2_T*_x_* _,_Pd/APS-Ti_3_C_2_T*_x_*_._

**Figure S10**

**
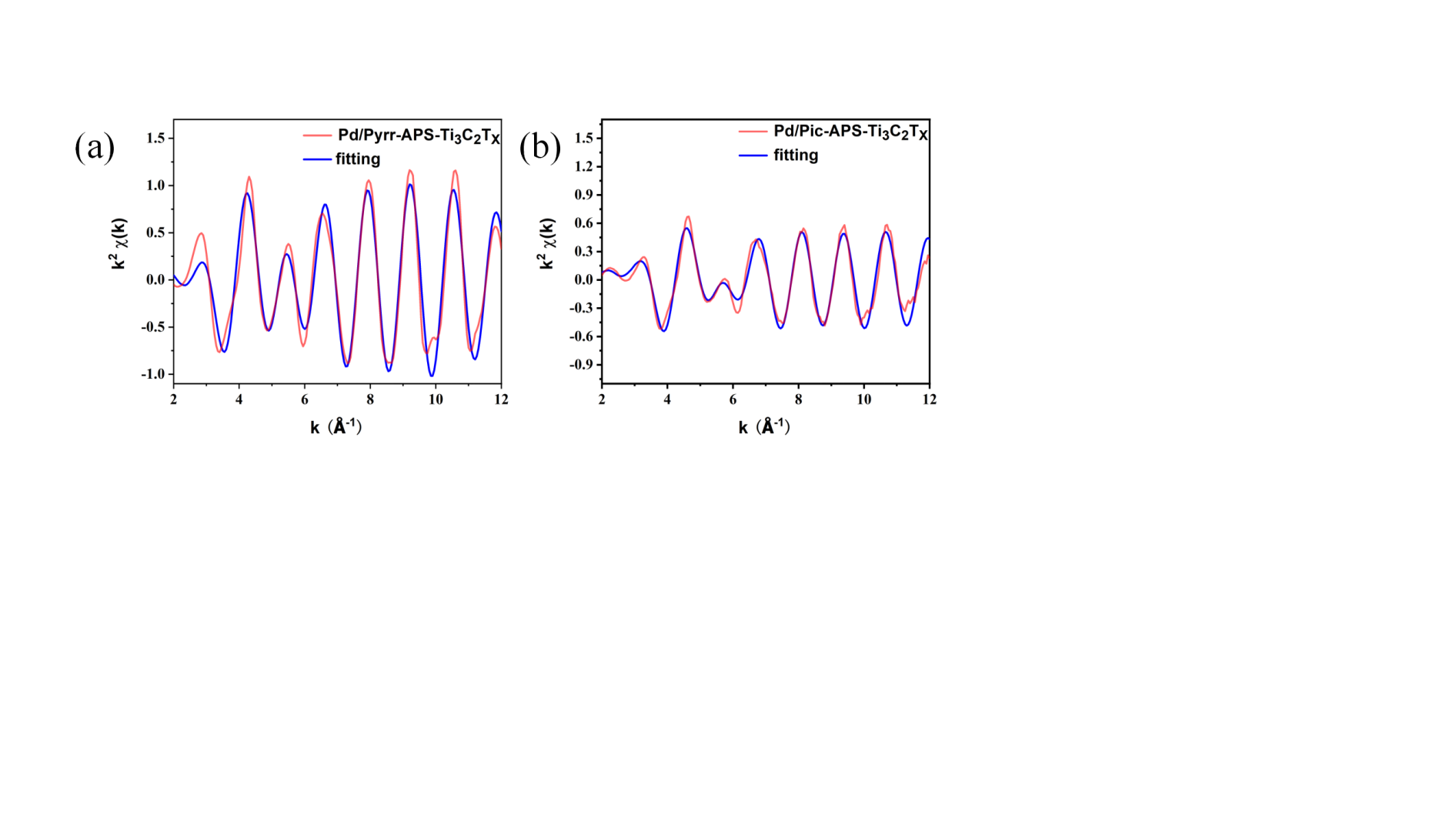
**

**Figure S10** EXAFS *R* space fitting curves for: a) Pd/Pyrr-APS-Ti_3_C_2_T*_x_*, and b) Pd/Pic-APS-Ti_3_C_2_T*_x_*_._

**Figure S11**


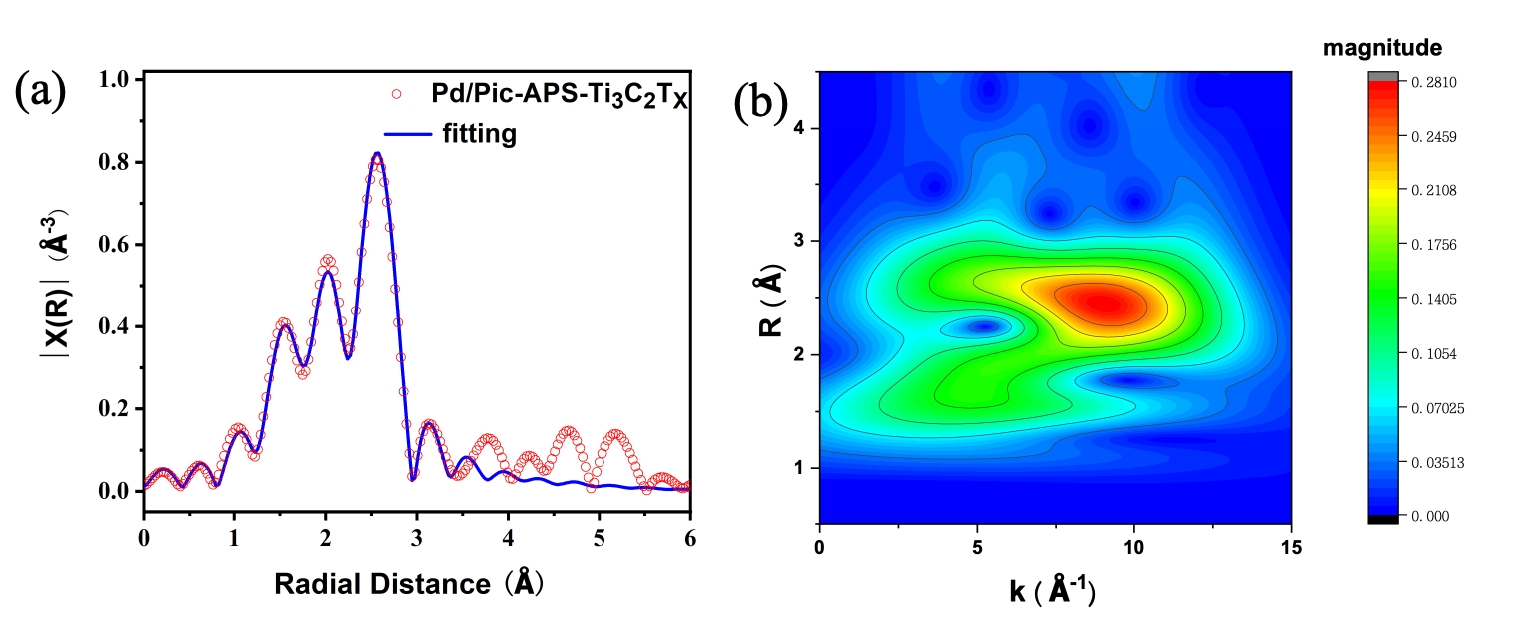


**Figure S11** EXAFS *k* space fitting curves and wavelet transforms of Pd/Pic-APS-Ti_3_C_2_T*_x_*_._

**Figure S12**


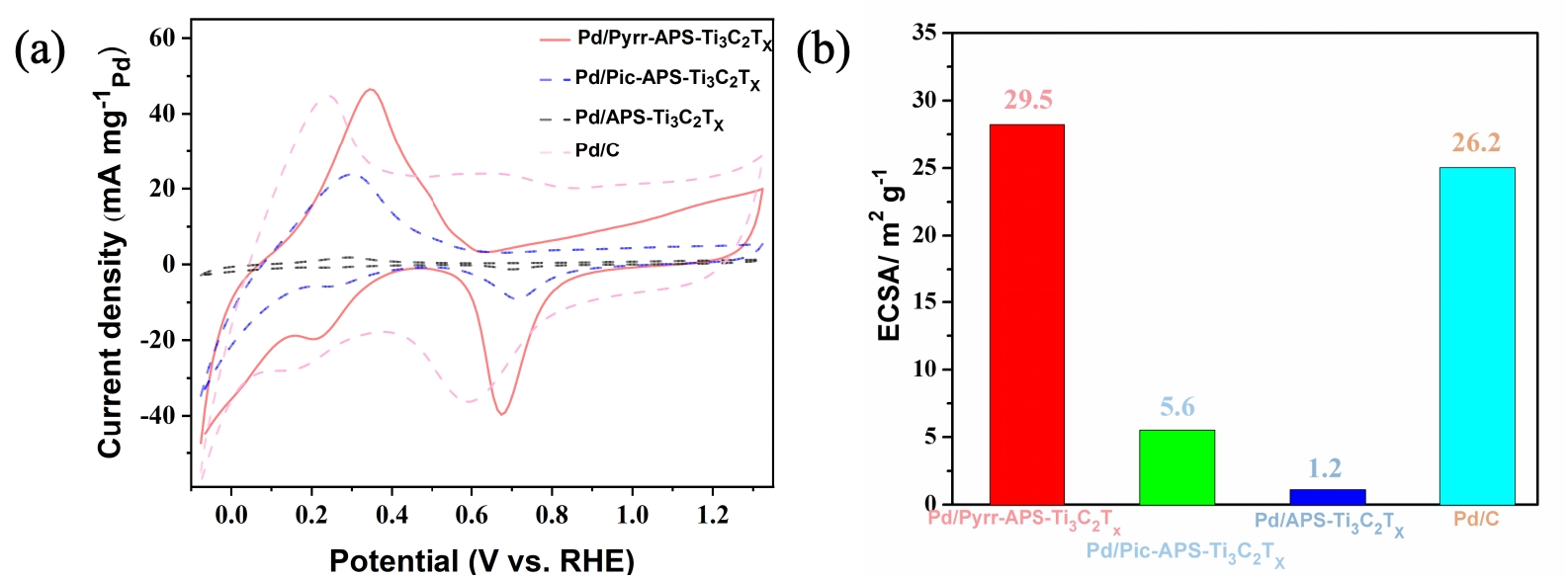


**Figure S12** a) CV curves at 1 m KOH at 50 mV s^−1^; b) ECSA values for Pd/Pyrr-APS-Ti_3_C_2_T*_x_*, Pd/Pic-APS-Ti_3_C_2_T*_x_*, Pd/APS-Ti_3_C_2_T*_x_* and Pd/C.

**Figure S13**


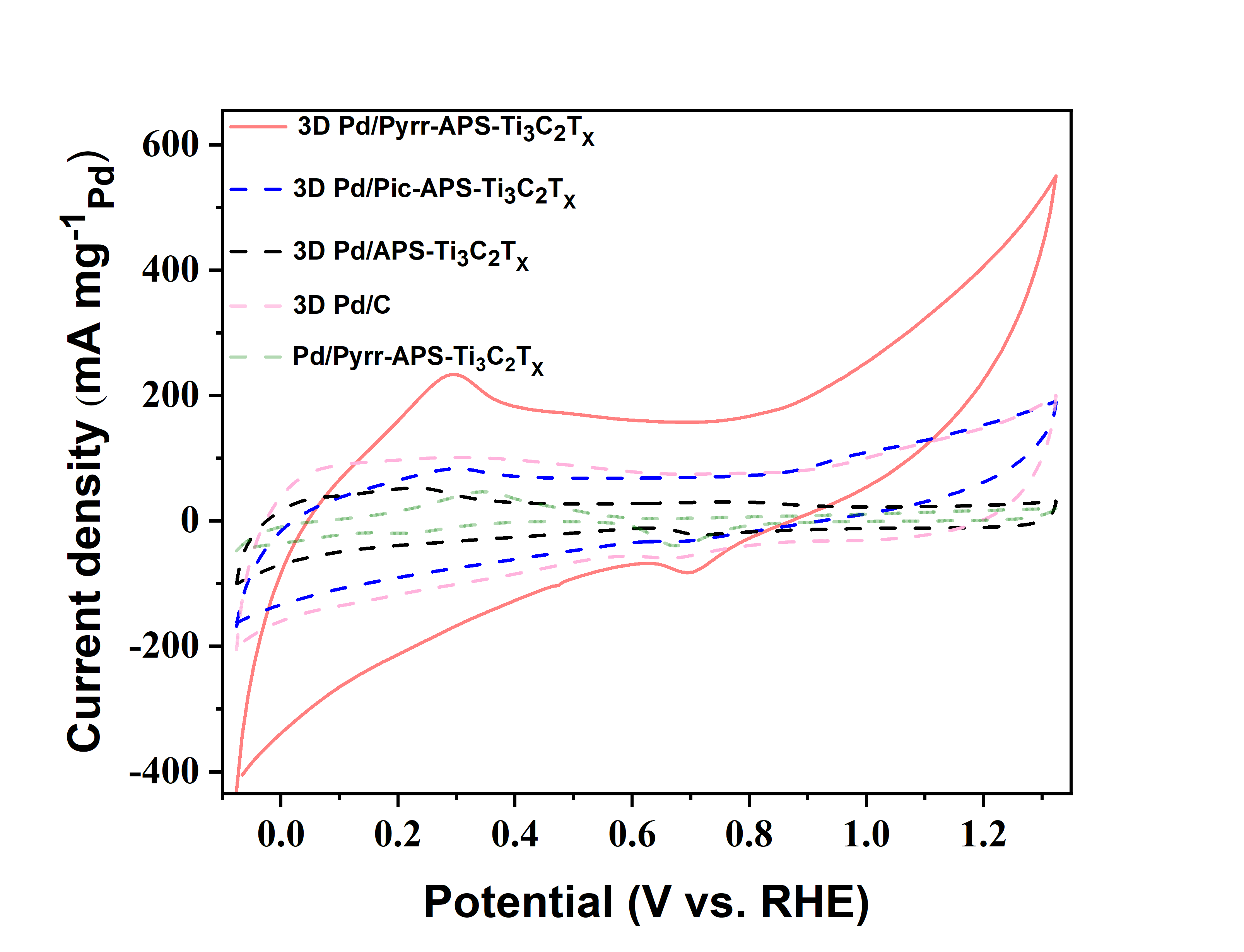


**Figure S13** CV curves at 1 m KOH at 50 mV s^−1^ for 3D Pd/Pyrr-APS-Ti_3_C_2_T*_x_*, 3D Pd/Pic-APS-Ti_3_C_2_T*_x_*, 3D Pd/APS-Ti_3_C_2_T*_x_* and 3D Pd/C.

**Figure S14**


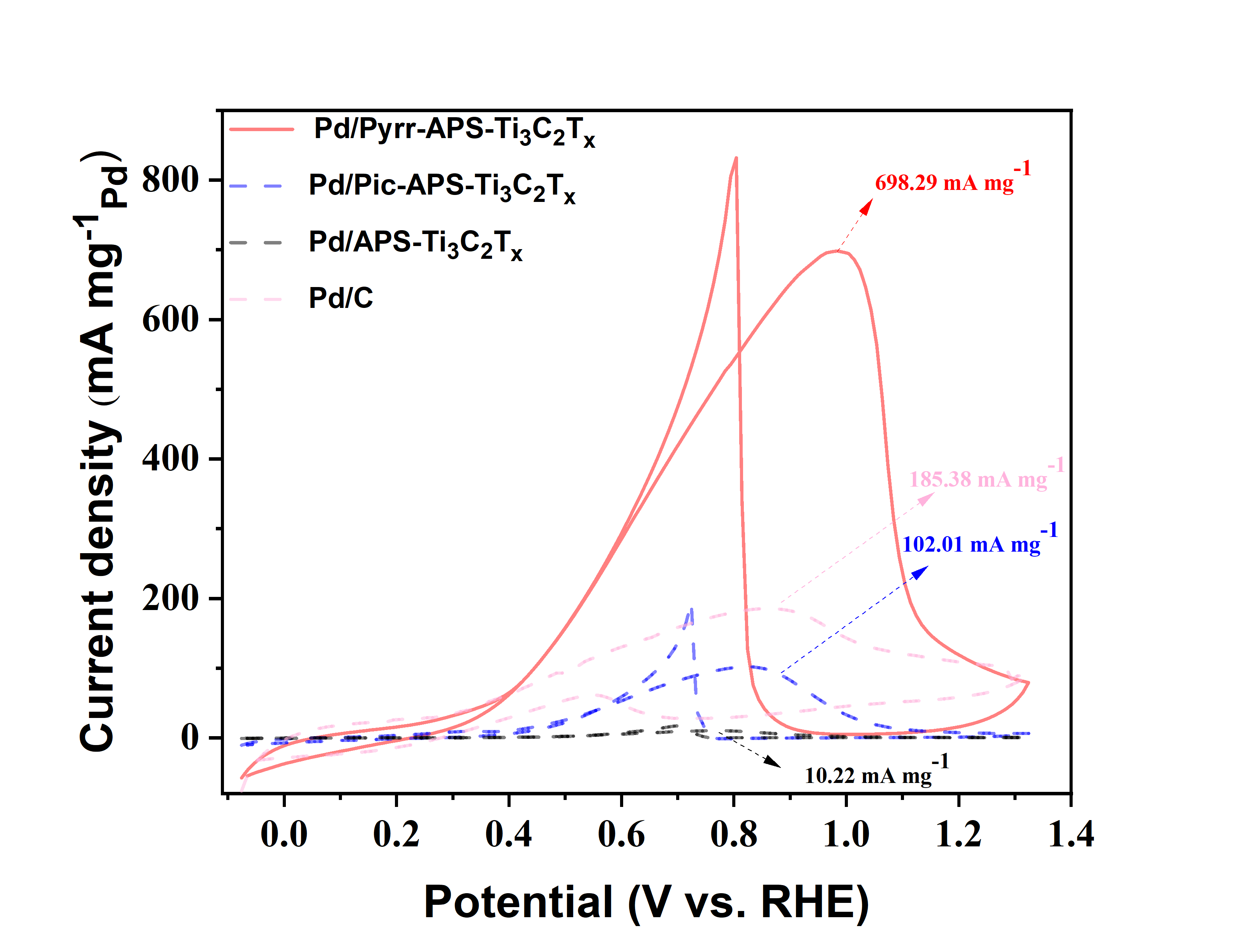


**Figure S14** CV curves at 1 m C_2_H_5_OH/KOH at 50 mV s^−1^ for powder Pd/Pyrr-APS-Ti_3_C_2_T*_x_*, Pd/Pic-APS-Ti_3_C_2_T*_x_*, Pd/APS-Ti_3_C_2_T*_x_* and Pd/C.

**Figure S15**


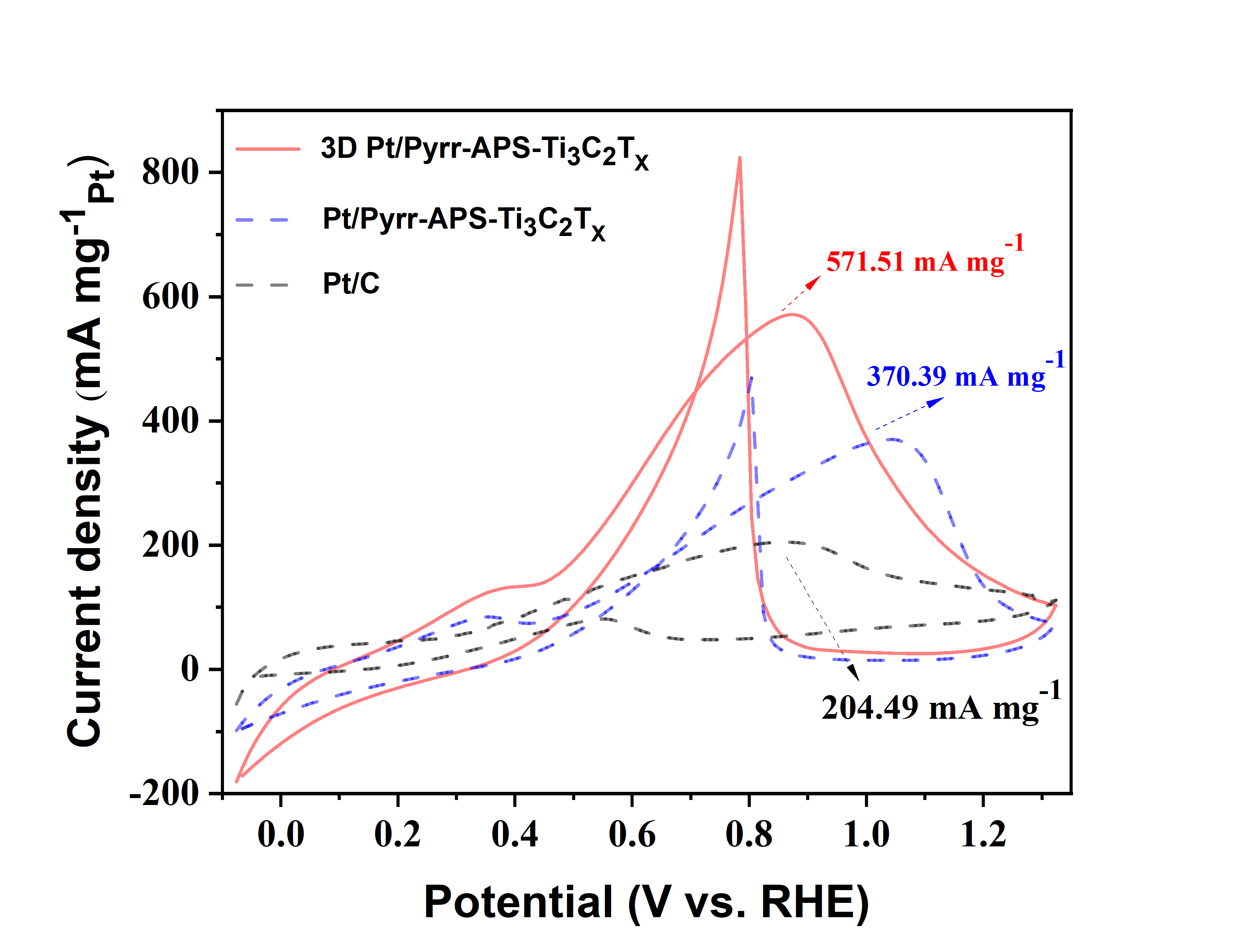


**Figure S15** CV curves at 1 m C_2_H_5_OH/KOH at 50 mV s^−1^ for 3D Pt/Pyrr-APS-Ti_3_C_2_T*_x_,* Pt/Pyrr-APS-Ti_3_C_2_T*_x_*, and Pt/C.

**Figure S16**


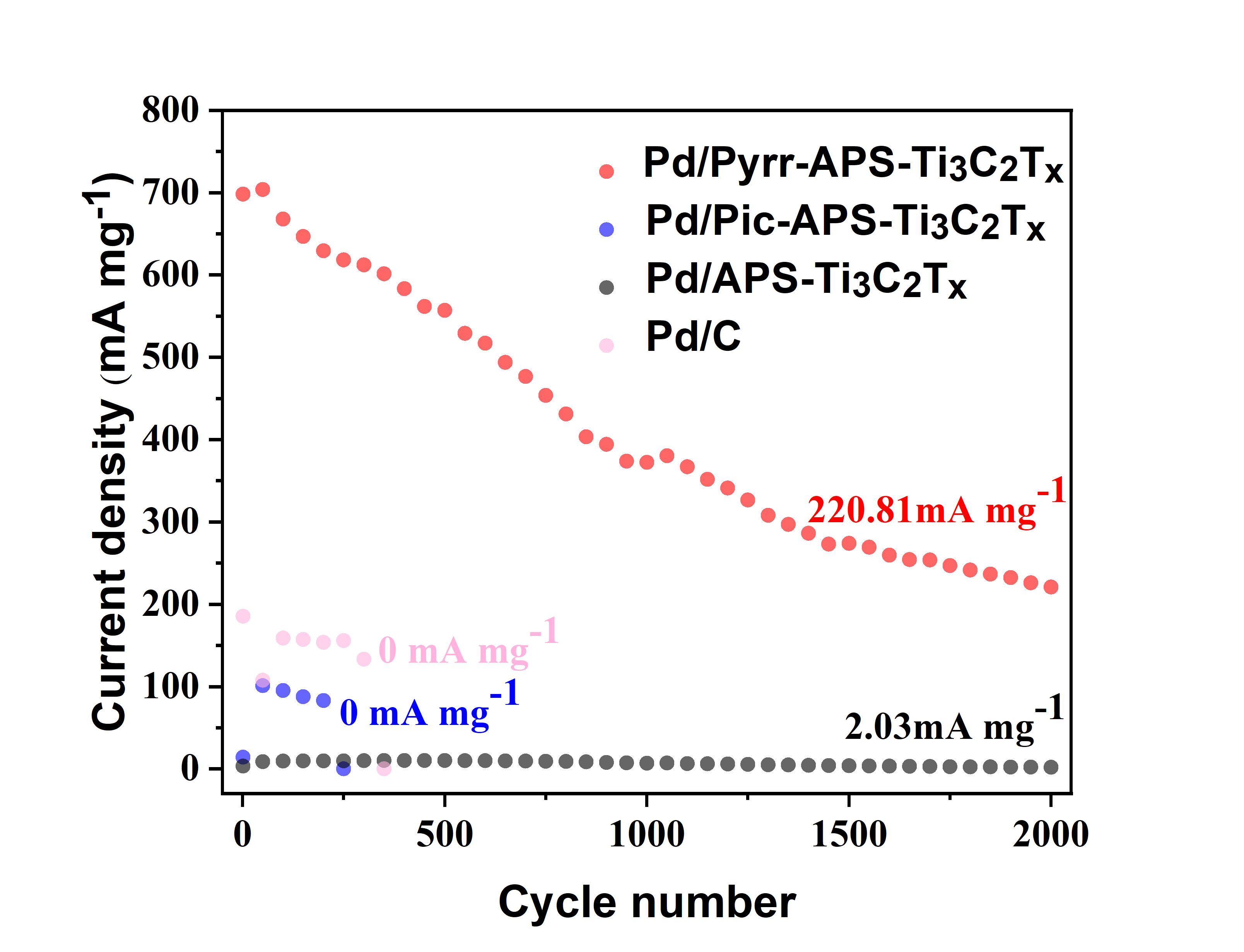


**Figure S16** Long-term durability of powder Pd/Pyrr-APS-Ti_3_C_2_T*_x_*, Pd/Pic-APS-Ti_3_C_2_T*_x_*, Pd/APS-Ti_3_C_2_T*_x_* and Pd/C at 1 m C_2_H_5_OH/KOH for 2000 cycles at 50 mV s^−1^.

**Figure S17**


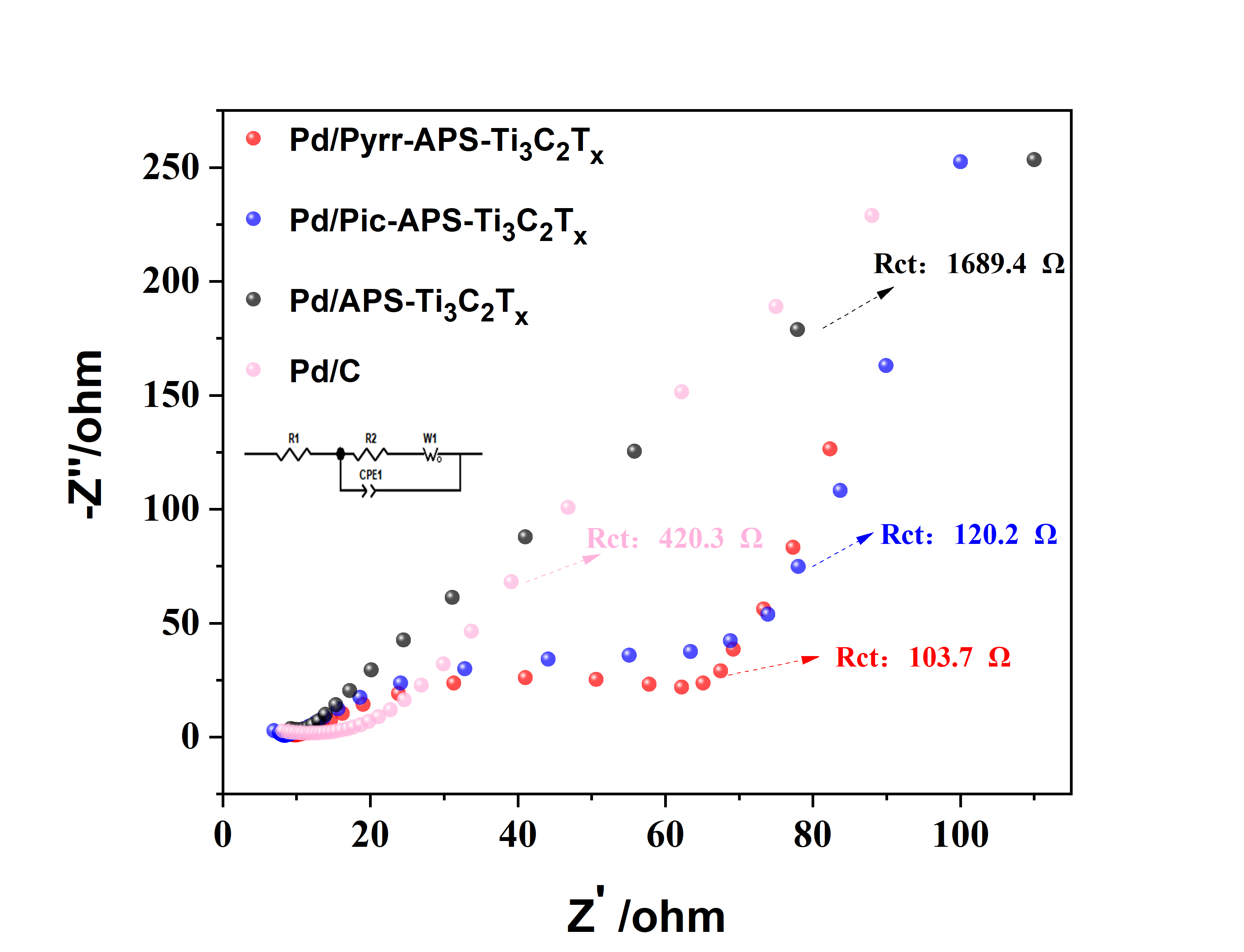


**Figure S17** EIS *Z*′/-*Z*″ plots of powder Pd/Pyrr-APS-Ti_3_C_2_T*_x_*, Pd/Pic-APS-Ti_3_C_2_T*_x_*, Pd/APS-Ti_3_C_2_T*_x_* and Pd/C.

**Figure S18**


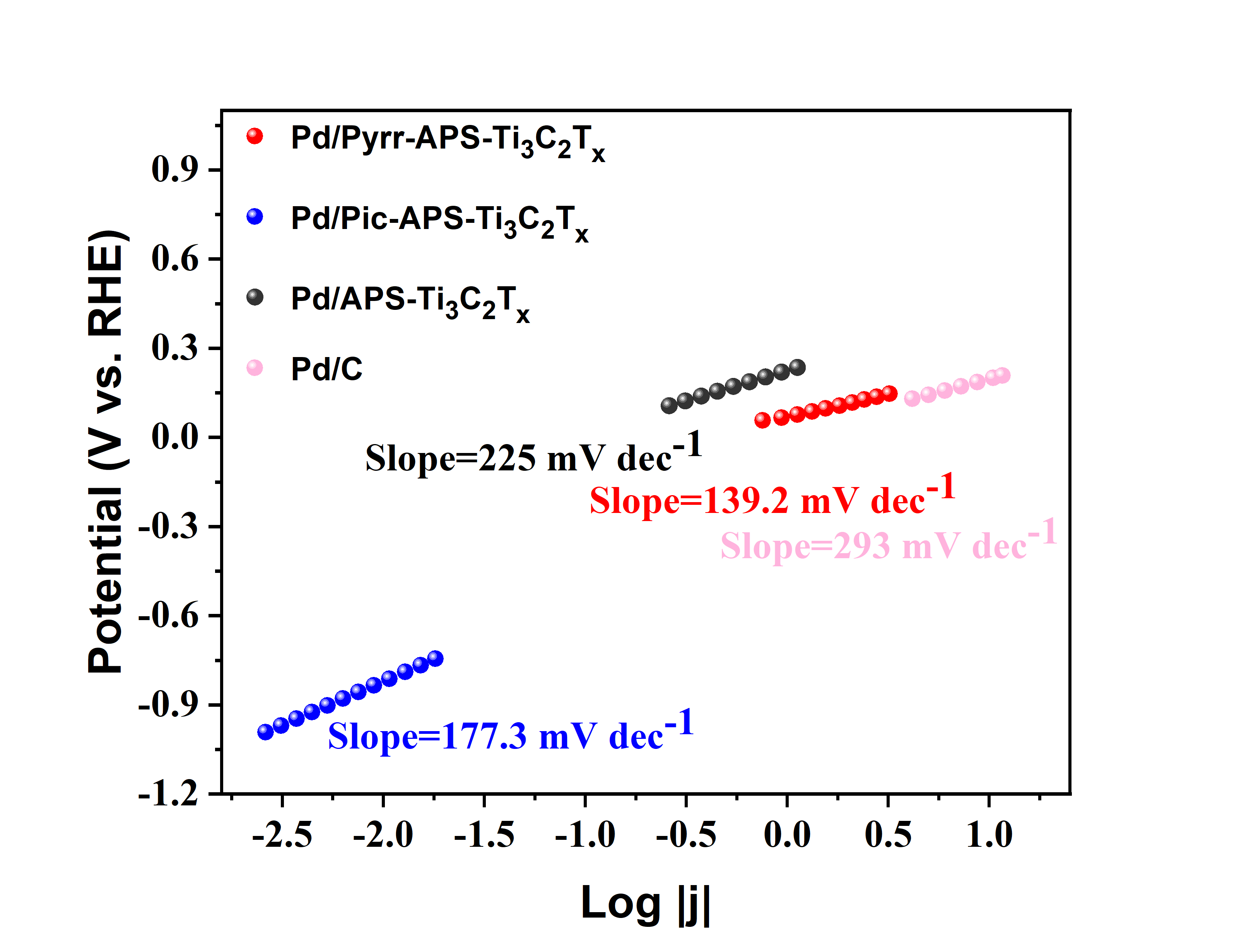


**Figure S18** Tafel slopes for powder Pd/Pyrr-APS-Ti_3_C_2_T*_x_*, Pd/Pic-APS-Ti_3_C_2_T*_x_*, Pd/APS-Ti_3_C_2_T*_x_* and Pd/C.

**Figure S19**

**
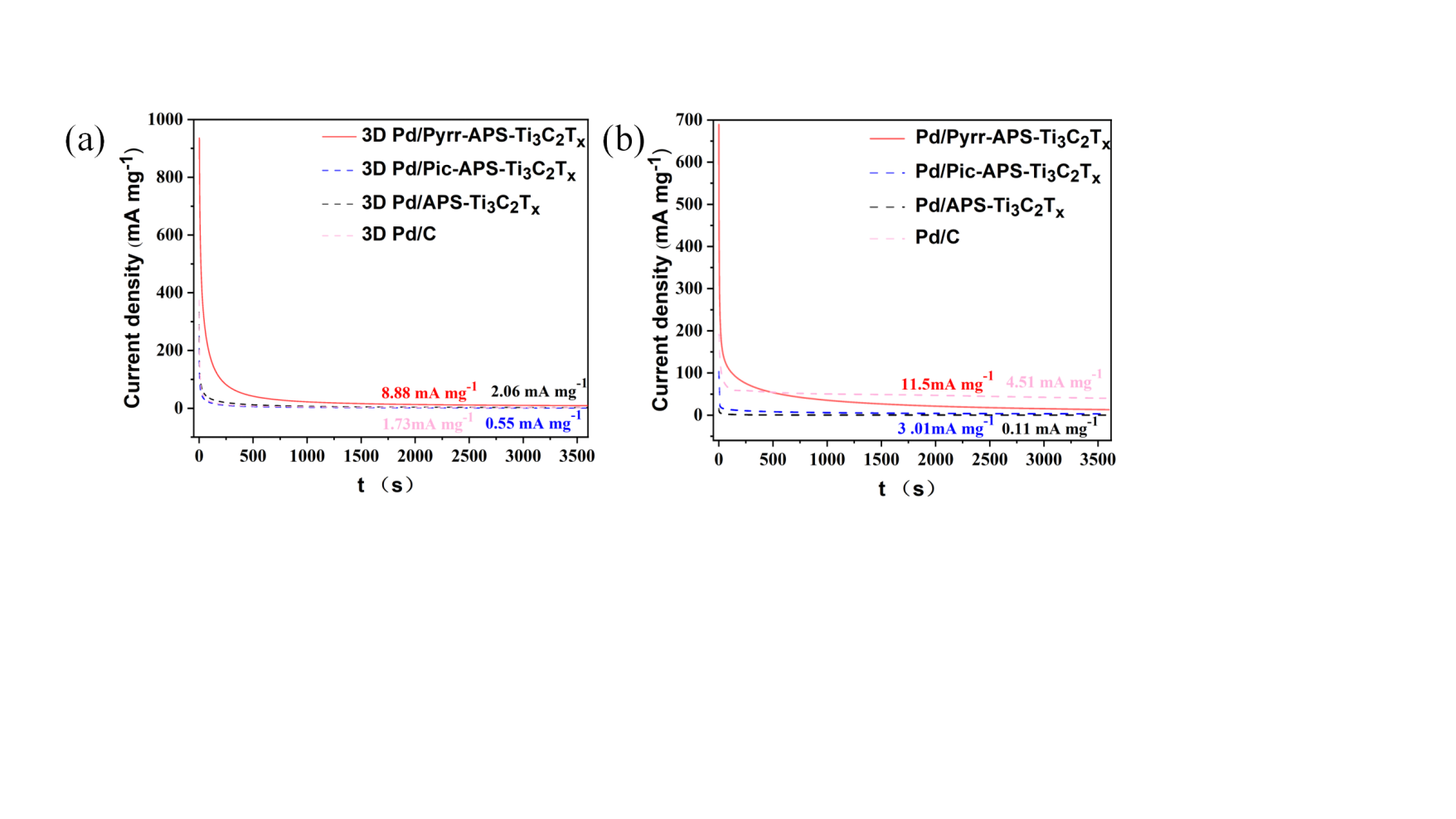
**

**Figure S19**  Chronoamperometry curves for a) the four 3D electrodes; b) the four powder electrodes at 0.9 V vs RHE for 1h.

**Figure S20**


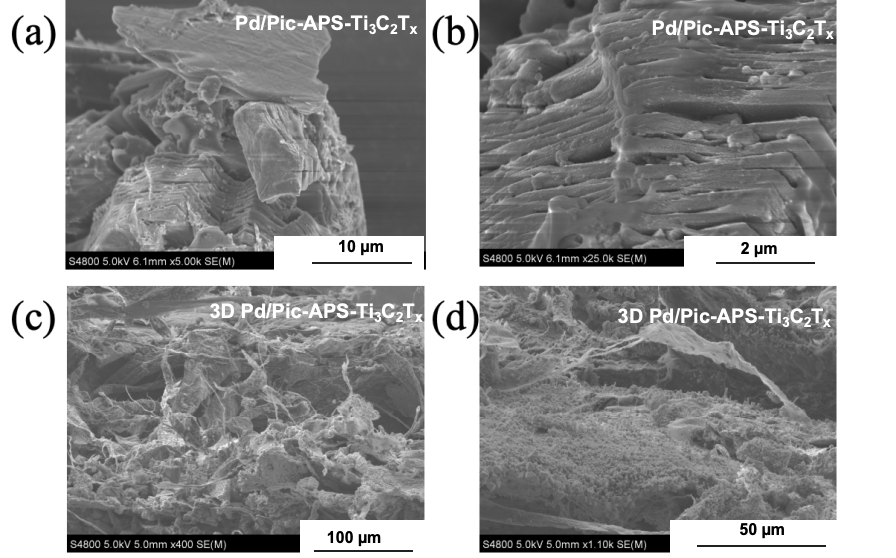


**Figure S20** SEM images of: **(**a), (b) powder Pd/Pic-APS-Ti_3_C_2_T*_x_* and
(c), (d) 3D Pd/Pic-APS-Ti_3_C_2_T*_x_* after 2000 cycles.

**Figure S21**


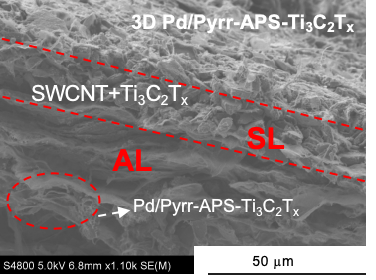


**Figure S21** SEM image of 3D Pd/Pyrr-APS-Ti_3_C_2_T*_x_* after 10000 cycles.

**Figure S22**


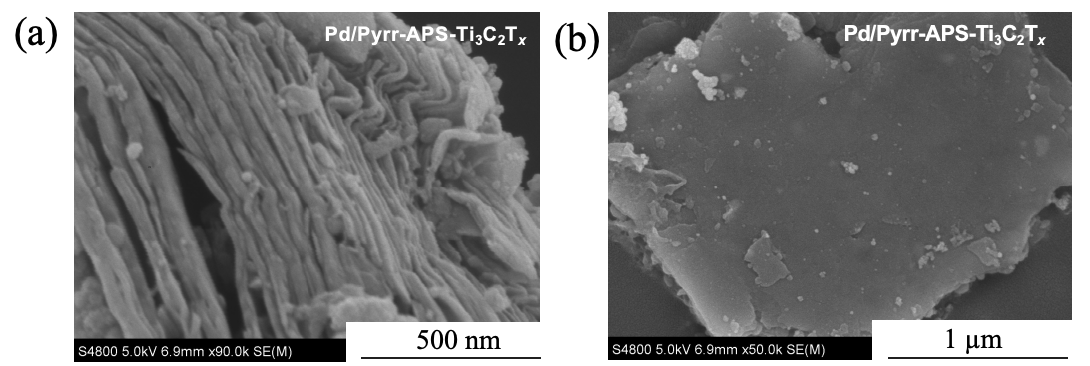


**Figure S22** SEM images (at different magnifications) of powder Pd/Pyrr-APS-Ti_3_C_2_T*_x_* after 2000 cycles.

**Figure S23**


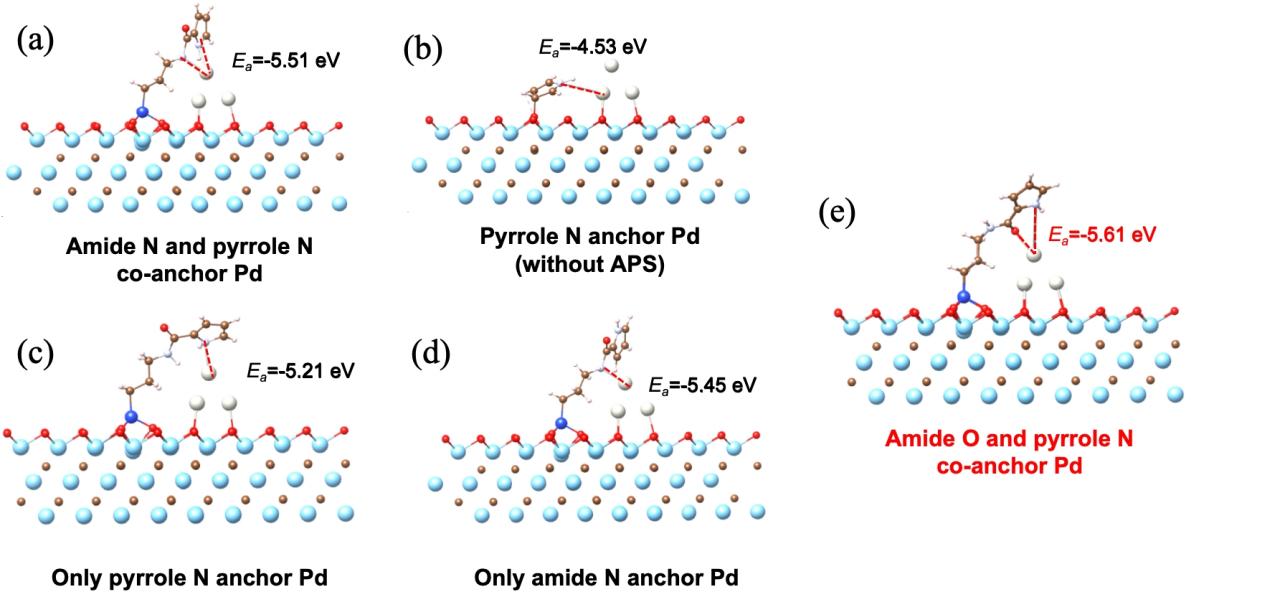


**Figure S23** Adsorption energies of Pd in Pd/Pyrr-APS-Ti_3_C_2_T*_x_* for various ligation combinations.

**Figure S24**


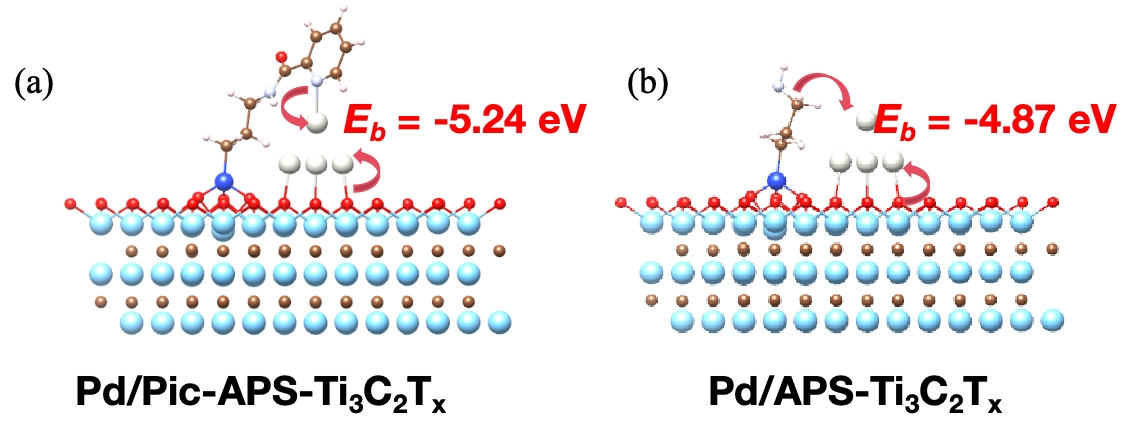


**Figure S24** Adsorption energies of Pd and the support in Pd/Pic-APS-Ti_3_C_2_T*_x_* and Pd/APS-Ti_3_C_2_T*_x_*.

**Figure S25**


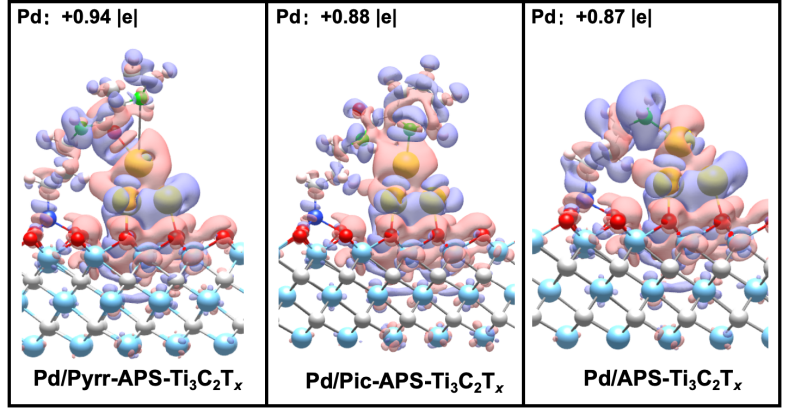


**Figure S25** Calculated differential charge density of Pd atoms in the three novel catalysts.

**Figure S26**


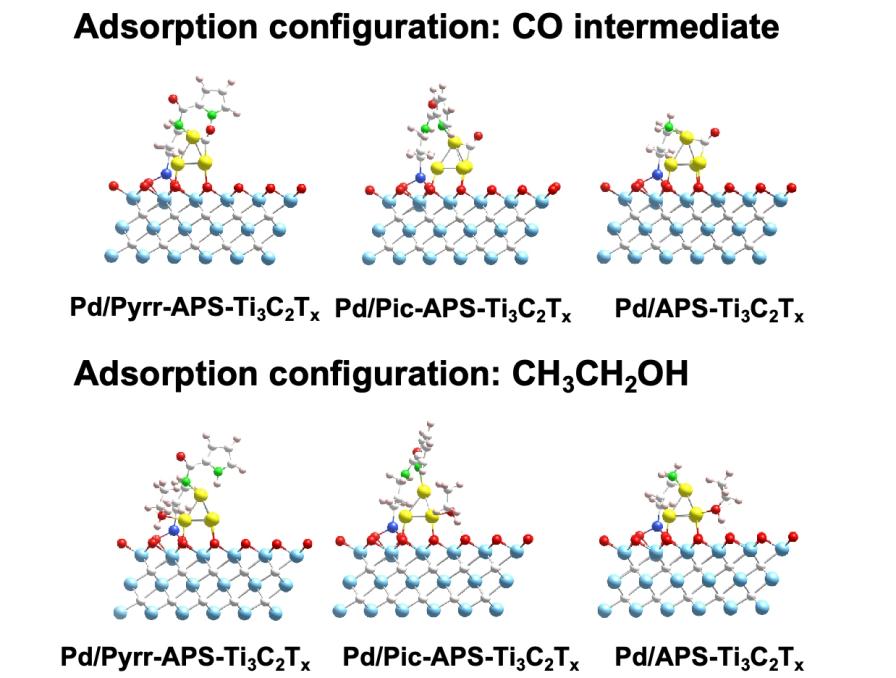


Figure S26 DFT-calculated models with the configurations of adsorbed species during EOR stages for Pd/Pyrr-APS-Ti_3_C_2_T*_x_*, Pd/Pic-APS-Ti_3_C_2_T*_x_* and Pd/APS-Ti_3_C_2_T*_x_*.
